# Warm Autoimmune Haemolytic Anaemia Management in Asia‐Pacific: A Delphi Panel Expert Consensus and Systematic Literature Review

**DOI:** 10.1002/jha2.70174

**Published:** 2025-11-06

**Authors:** Phil Choi, Alberta Hoi, Rong Fu, Jun‐ho Jang, Yok Lam Kwong, Yoshitaka Miyakawa, Chi‐Chiu Mok, Hiroaki Niiro, Yasutaka Ueda, Robert Bagnall, Petya Kodjamanova, Da Eun Ahn, Nakul Saxena, Bryan Wahking, Shin‐Seok Lee

**Affiliations:** ^1^ Caberra Hospital Canberra Australia; ^2^ Department of Rheumatology Monash Health School of Clinical Sciences Monash University Melbourne Australia; ^3^ Department of Hematology General Hospital Tianjin Medical University Tianjin China; ^4^ Samsung Medical Center Seoul South Korea; ^5^ The University of Hong Kong Hong Kong China; ^6^ Tianjin Medical University General Hospital Tianjin China; ^7^ Saitama Medical University Morohongo Japan; ^8^ Tuen Mun Hospital Tuen Mun Hong Kong China; ^9^ Kyushu University Kyushu Japan; ^10^ Osaka University Osaka Japan; ^11^ Health Economics and Market Access Amaris Consulting Barcelona Spain; ^12^ Health Economics and Market Access Amaris Consulting London UK; ^13^ Johnson & Johnson Innovative Medicines Singapore Singapore; ^14^ Chonnam National University Medical School & Hospital Donggu Gwangju South Korea

**Keywords:** clinical burden, economic burden, epidemiology, warm autoimmune haemolytic anaemia

## Abstract

**Objective:**

This study aimed to assess the burden of warm autoimmune haemolytic anaemia (wAIHA) and treatment practices in the Asia‐Pacific (APAC) region.

**Methods:**

A systematic literature review (SLR) of observational studies in adult patients with wAIHA was conducted. Based on these findings, a Delphi panel was convened to develop consensus recommendations on diagnosis, treatment goals and algorithms by subtypes, unmet needs and research priorities.

**Results:**

The SLR included 37 publications. Clinical guidelines consistently recommend direct antiglobulin testing for diagnosis and corticosteroids as first‐line treatment. Real‐world data showed corticosteroid use in over 80% of patients, with response rates of 81%–96%, and relapse rates of 18%–45%. The Delphi panel recommended routine screening for underlying conditions and early therapeutic interventions. Key challenges identified included restricted access to rituximab in parts of the APAC region and gaps in physician awareness. Research priorities included optimising therapy, assessing long‐term outcomes and improving disease awareness.

**Conclusion:**

This research emphasises the need for evidence‐based, regionally adaptable treatment algorithms for wAIHA in the APAC region. Ongoing data gaps related to epidemiology, outcomes and quality of life must be addressed to enhance patient care.

**Trial Registration:**

The authors have confirmed clinical trial registration is not needed for this submission.

## 1 Introduction

Autoimmune haemolytic anaemia (AIHA), a rare disorder characterised by the destruction of erythrocytes due to autoantibodies, is typically diagnosed by the presence of haemolytic anaemia and a positive direct antiglobulin test (DAT). Using global data, the estimated incidence and prevalence of AIHA are 0.8–3 per 100,000 persons annually and 6–21 per 100,000 persons, respectively [1]. AIHA can be classified as either primary (idiopathic) or secondary to underlying malignancies, immunological disorders, infections or medications [2, 3, 4]. Uncontrolled AIHA is life‐threatening due to thromboembolisms, infections and other related causes [5]. The presentation and severity of AIHA depend primarily on patient comorbidities, functional status and immune system integrity, as well as underlying diseases [1, 6]. International guidelines recommend corticosteroids as the first‐line treatment for AIHA, and other immunosuppressive therapies and biologics, such as rituximab, as subsequent options [4].

Warm AIHA (wAIHA) is the most common AIHA subtype, accounting for 60%–80% of cases [2, 3]. It is characterised by the presence of immunoglobulin G (IgG) autoantibodies that are optimally active at 37°C. The condition predominantly affects females, has a median age of onset of 68.7 years, and is secondary in approximately 50% of cases [7], most often due to systemic lupus erythematosus (SLE) and other subtypes of connective tissue disease (CTD).

Despite its clinical importance, information on wAIHA in the Asia‐Pacific (APAC) region is limited. To address this, we conducted a systematic literature review (SLR) to evaluate the burden across epidemiological, clinical and economic dimensions, followed by a Delphi panel of APAC experts to build consensus on diagnosis, treatment and research priorities for the region.

## 2 Methods

### 2.1 Systematic Literature Review Methodology

The reporting of this SLR was guided by the Preferred Reporting Items for Systematic Review and Meta‐Analysis (PRISMA) [8].

#### 2.1.1 Search Strategy

China National Knowledge Infrastructure (CNKI), MEDLINE, MEDLINE‐IN‐PROCESS, EMBASE, ICHUSHI, KOREAMED and WANFANG databases were searched for studies published from database inception to 12 October 2022 (the search date) using MeSH/EMTREE and free‐text terms (Tables S1–S5). The proceedings of relevant congresses (Table S6) and Google Scholar were searched for relevant abstracts and studies. The searches included combinations of: ‘waiha OR warm autoimmune haemolytic anaemia,’ ‘epidemiology,’ ‘socioeconomics,' and ’quality adjusted life year.

#### 2.1.2 Study Selection

Duplicate records were removed using Zotero (Corporation for Digital Scholarship). Two reviewers independently screened titles and abstracts against pre‐defined eligibility criteria for inclusion (below). Full‐text articles assessed as potentially relevant during title and abstract screening were screened by two reviewers. At each screening step, differences were resolved by discussion with a third reviewer. A list of excluded studies with the reason for exclusion is presented in Table S7.

Pre‐defined study selection criteria are summarised in Table 1 according to the PICOS (Population, Intervention, Comparator, Outcome and Study type) framework [9]. Observational studies on the epidemiology or clinical, patient or economic burden of adult patients with AIHA or wAIHA in APAC countries published in Chinese, Japanese, Korean or English since 2012 were included. Reviews and clinical practice guidelines were also included for cross‐referencing.

**TABLE 1:** PICOS framework.

Criteria	Description
Population	Adult patients with wAIHA. Subgroups of interest included, but were not limited to: Patients with primary wAIHA Patients with secondary wAIHA Patients with underlying conditions Patients with different disease severity
Interventions and comparators	No restriction (but precedence was given to pharmacological treatments)
Outcomes	**Epidemiology** Data on the prevalence and incidence of wAIHA and subgroups of interest was targeted. However, where this was not available, the prevalence and incidence of AIHA was sought, and if possible/available, the proportion of patients with AIHA who were classified as wAIHA was noted. Data on the mortality outcomes of patients with w/AIHA was also targeted. **Clinical burden** The burden of symptoms experienced by patients with wAIHA, the proportion of patients who were dependent on RBC transfusions, the morbidity experienced by patients and the risk factors associated with the development of wAIHA. **Patient burden** Patient burden as demonstrated via EQ‐5D, SF‐36, WHOQOL‐BREF, VAS, other relevant/identified scales or any other way of reporting patient burden. **Economic burden** The direct, including total, inpatient, outpatient and other direct costs, and indirect, including productivity losses, absenteeism and other indirect costs, costs and resource use associated with wAIHA and its management.
Study type	Observational studies, reviews^a^ (case reports, letters, and news articles were not included)
Language	Mandarin, English, Japanese, Korean
Publication date	Studies published since 2012

^a^Reviews were included in this review for cross‐referencing purposes.

Abbreviations: E5QD: EuroQol Five‐Dimension Scale Questionnaire; RCT: randomised controlled trial; SF‐36: Short Form (36) Health Survey; VAS: visual analogue scale; (w) AIHA: (warm) autoimmune haemolytic anaemia; WHOQOL‐BREF‐ World Health Organization Quality of Life‐BREF.

#### 2.1.3 Data Extraction

Data were extracted using a pre‐designed Microsoft Excel tabular summary, capturing publication details, study design, patient characteristics and outcomes related to epidemiology and clinical, patient and economic burden. Twenty percent of arbitrarily selected articles were reviewed by a third reviewer. Results from the SLR were analysed descriptively without statistical testing.

### 2.2 Delphi Panel Methodology

Rheumatologists and haematologists from APAC experienced in wAIHA management and proficient in English were recruited to participate in the Delphi panel. Candidate panellists were contacted sequentially in order from most to fewest peer‐reviewed publications until ten experts were enrolled. Panellists completed three rounds of anonymised electronic questionnaires (Appendix S2). Consensus for each question, predefined as ≥ 80% agreement, was assessed after each round [10].

## 3 Results

### 3.1 Systematic Literature Review

#### 3.1.1 Characteristics of Identified Studies

**FIGURE 1:**
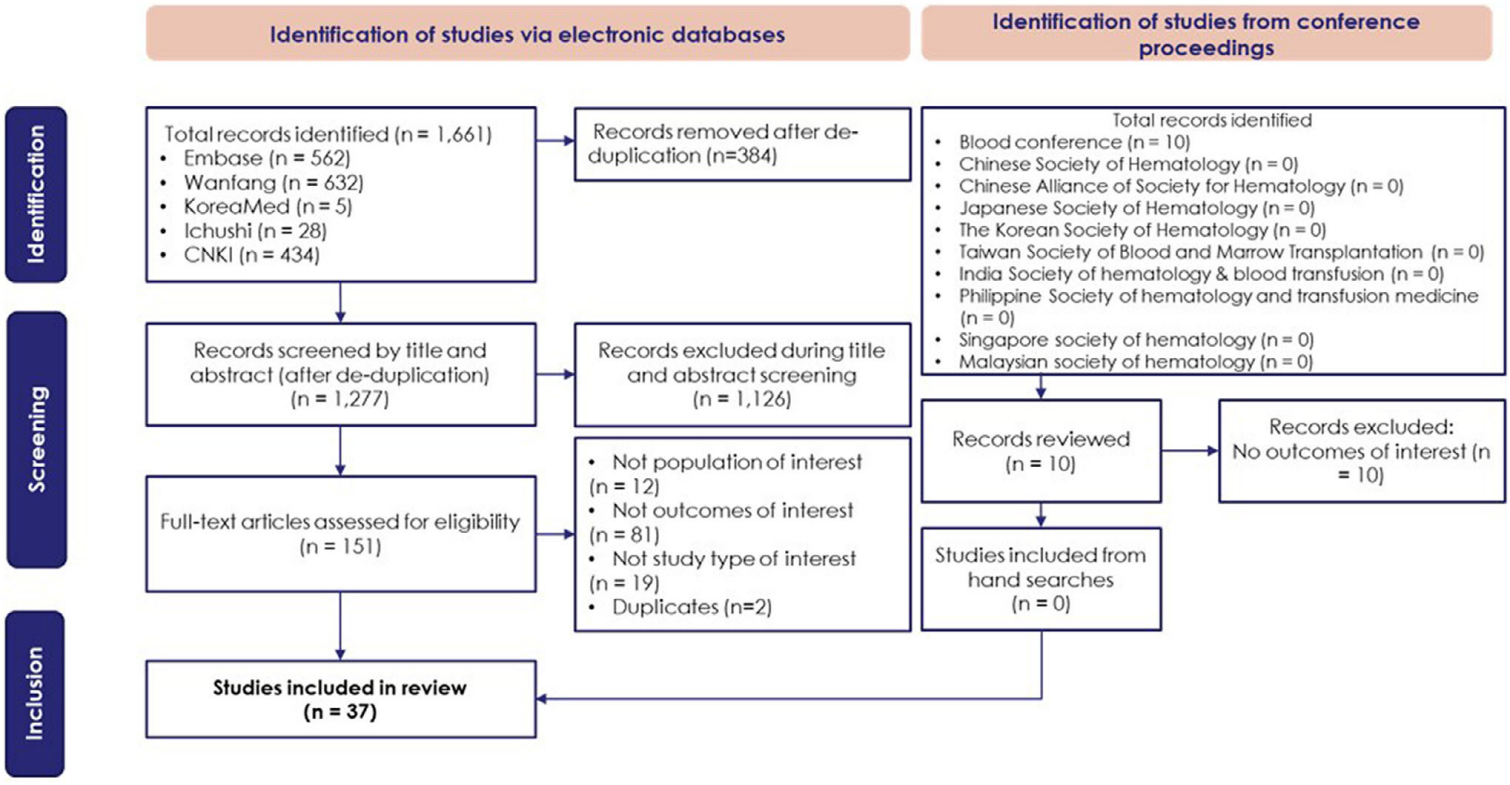
PRISMA diagram.

The electronic search yielded 1661 records, of which 1277 were screened after removing duplicates. Following full‐text review of 151 articles, 37 studies were included (Figure 1). Most studies meeting the inclusion criteria were conducted in China (*n* = 26), with others performed in Japan (*n* = 4), India (*n* = 3), South Korea (*n* = 2), Thailand (*n* = 1) and Taiwan (*n* = 1) (Table S8). Clinical outcomes were most frequently reported (*n* = 25), followed by epidemiology (*n* = 13) and economic burden (*n* = 1); no studies addressed quality of life. Study designs included retrospective (*n* = 22), prospective (*n* = 10) and cross‐sectional (*n* = 1), and four studies with other designs.

#### 3.1.2 AIHA Epidemiology and Mortality in APAC

Two studies reported population‐wide incidence/prevalence. In Japan, AIHA incidence is 1–5 per million and prevalence 3–10 per 1 million [11]. In Taiwan, incidence was 23.76 per 100,000 person‐years among people living with human immunodeficiency virus and 0.84 per 100,000 person‐years among age‐ and sex‐matched controls [12]. Most patients with AIHA were female (60%–79%) [13, 14, 15, 16, 17, 18, 19]. Predominantly, AIHA cases were classified as wAIHA (47%–95%) [11, 13, 14, 15, 16, 17, 18, 19, 20, 21, 22, 23, 24] and were secondary (56%–90%) [16, 18, 21, 24, 25, 26], most commonly to cancer, infections, medications and autoimmune diseases [4, 25, 26, 27, 28, 29, 30, 31, 32]. Survival was generally good (70% 10‐year survival in Japan; 84% 5‐year survival in Taiwan) [31, 33], but worse in secondary AIHA and patients with DAT‐negative or IgA‐mediated AIHA [23, 31, 34]. Malignancies were the main cause of death among patients with secondary AIHA (55%) [31].

#### 3.1.3 Diagnostic Testing

Our search strategy identified clinical practice guidelines from China, Japan and South Korea offering AIHA and wAIHA diagnostic recommendations [33, 35, 36]. Guidelines recommend diagnosing AIHA based on haemolytic anaemia and a positive DAT. Diagnostic indicators include low haemoglobin (Hb) and high reticulocyte count, bilirubin and lactate dehydrogenase (LDH). Guidance in China and Japan also includes low haptoglobin [33], and South Korea includes spherocytosis [36]. DAT is the primary recommended diagnostic test, with additional subtype‐specific tests including indirect antiglobulin test (IAT), cold agglutinin and Donath–Landsteiner (D‐L). In addition, guidelines in South Korea recommend enhanced DAT techniques to avoid false negatives [36].

#### 3.1.4 Treatment Recommendations

Clinical practice guidelines from China, Japan and South Korea recommend corticosteroids as frontline AIHA and wAIHA treatment [33, 35, 36]. All guidelines provide splenectomy and immunosuppressive drugs (e.g., cyclophosphamide, azathioprine [AZA], vinblastine, danazol, cyclosporine A or mycophenolate mofetil [MMF]) as second‐line treatments. Guidelines from China and South Korea note rituximab as a second‐line option, while guidelines from Japan provide no specific recommendations for its use. Guidance from China notes that blood transfusions should only be given to patients with acute haemolytic anaemia and to patients with chronic anaemia with Hb < 50 g/L.

#### 3.1.5 Treatment Patterns and Outcomes

Across the APAC region, frontline AIHA and wAIHA treatment has relied on corticosteroids. Almost all patients (> 80%) received corticosteroids, typically in the first line of treatment [16, 19, 25, 28, 31, 37], with 81%–96% initial response rates [17, 19, 31]. However, 18%–45% relapsed within a year [17, 31]. Second‐line treatments included immunosuppressants, rituximab, IVIG and occasionally splenectomy or plasmapheresis [16, 19, 25, 31, 37, 38]. Three studies reported that rituximab improved clinical outcomes versus corticosteroids, even at low doses [39, 40, 41]. Blood transfusions were commonly used (28%–60% in AIHA [16, 28, 42], 47.7–67.5% in wAIHA) [27, 29, 43], and were generally effective. Transfusions relieved anaemia with minimal adverse events [27, 44].

#### 3.1.6 Clinical Burden

About 40% of patients with wAIHA had severe haemolysis, often due to delayed diagnosis. Most patients experienced fatigue, pallor, dyspnoea and dark urine [41, 45]. In another study, over half of the patients (55.1%) were transfusion‐dependent and some developed infections [39]. In the most recent study conducted in China, corticosteroid‐non‐responding patients with SLE‐AIHA receiving whole blood exchange alone had a shorter hospital stay (12.86 days) and lower costs ($4013.82) than those with second‐line IVIG or rituximab (18 days; $8147.77) [46]. Another study in China found that 16.7% (2/12) of patients with wAIHA experienced thrombosis and 50% (6/12) developed infections [37]. Thrombotic events are more common in AIHA due to factors such as free Hb from haemolysis, endothelial activation, inflammation and procoagulant microparticles [47, 48, 49]. Corticosteroids may further increase thrombotic risk, compounding the underlying prothrombotic state [50].

### 3.2 Delphi Panel

**FIGURE 2:**
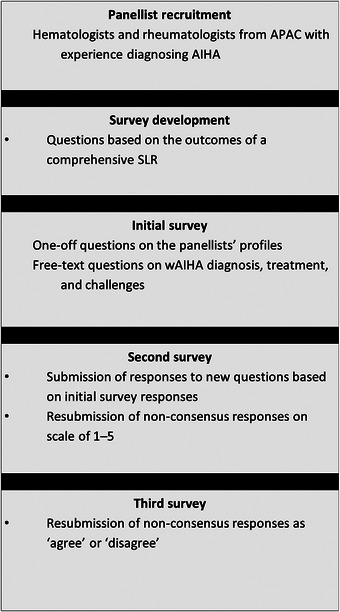
Summary of Delphi process.

Ten experts (three rheumatologists and seven haematologists) from Japan (*n* = 3), Australia (*n* = 2), Hong Kong (*n* = 2), South Korea (*n* = 2) and China (*n* = 1) participated in the Delphi panel (Figure 2). Nine panellists completed the first two rounds, while all ten panellists participated in round three. All panellists had experience diagnosing AIHA, primarily in adult patients. Responses are summarised in Tables S11–S25, with consensus statements summarised in Tables 2, 3, 4. Statements for which consensus was not reached are summarised in Table S9, and statements where there was a consensus disagreement are summarised in Table S10.

**TABLE 2:** Consensus statements related to wAIHA diagnosis.

Important screening tests aside from DAT	Proportion of panellists agreed
Assessment for possible underlying diseases (e.g., CLL, cancer, SLE, infection, drug use, other autoimmune diseases)	89%
Blood smear with spherocytes	89%
Rheumatoid factor test in patients who might have rheumatoid arthritis	90%

Optimal strategies for evaluating and addressing the underlying diseases that cause secondary wAIHA	Proportion of panellists agreed
Each rheumatic disease has its respective approach to evaluating disease activity and chronicity/ damage/ comorbidities of the individual	100%
Symptoms of possible underlying diseases and blood tests	90%
Clinical review (history, examination, and relevant investigations) with a low threshold to exclude lymphoproliferative disease	90%
The most common initial step is to evaluate connective tissue disease	100%
Rule out other haematologic disease	89%
Screen autoimmune diseases and malignancies	89%

Signs intervention is needed beyond ‘watch and wait’	Proportion of panellists agreed (%)
Hb level	100%
Symptoms of anaemia / symptomatic anaemia	100%
Severity of anaemia	89%
Hb under 10 mg/dL, if the patients feel anaemic symptoms	100%
Trajectory of falling Hb	100%
Underlying diseases that cause wAIHA	90%

Abbreviations: CLL: chronic lymphocytic leukaemia; Hb: haemoglobin; SLE: systemic lupus erythematosus; wAIHA: warm autoimmune haemolytic anaemia.

**TABLE 3:** Consensus statements related to wAIHA treatment.

Short‐term treatment goals for patients with wAIHA	Proportion of panellists agreed (%)
Improvement in blood count and increase Hb	100%
Improve anaemic symptoms and tiredness	100%
Stabilise Hb levels	80%
Remission	90%

Long‐term treatment goals for patients with wAIHA	Proportion of panellists agreed (%)
Cessation of treatment	90%
Improve Hb and quality of life, go back to work, to avoid side effects of corticosteroids	89%
Improvement of anaemia (Hb level)	100%
Improvement of symptoms (fatigue, etc.)	89%
Minimise steroid burden	90%
Prevent/reduce relapse	100%
Stabilise disease status	80%
Extinguish active haemolysis without relying on long‐term toxic therapies	100%
Cessation of anaemia treatment	89%
Prevention of complications (infections, thrombosis, etc.)	100%

First‐line treatments	Proportion of panellists agreed (%)
Corticosteroid: prednisone (1 mg/kg daily for 3 weeks)	100%
Corticosteroid: intravenous methylprednisolone (250–1,000 mg per day for 1–3 days)	80%

Second‐line treatments (after failure)	Proportion of panellists agreed (%)
Cyclosporin (2–5 mg/kg per day split into two doses)	89%
AZA (1–2 mg/kg/day split into multiple doses)	89%
Immunosuppressants	100%
Rituximab (1,000 mg given twice, 2 weeks apart) ± corticosteroids	80%
Rituximab (375 mg/m^2^ once a week for 4 weeks) ± corticosteroids	90%

Second‐line treatments after relapse	Proportion of panellists agreed (%)
Enrolment in clinical trials	80%
Immunosuppressants	89%
Cyclosporin (2–5 mg/kg per day split into two doses)	100%
AZA (1–2 mg/kg/day split into multiple doses)	100%
Rituximab (375 mg/m^2^ once a week for 4 weeks) ± corticosteroids	100%
Rituximab (1,000 mg given twice, 2 weeks apart) ± corticosteroids	90%

Third‐ or later‐line treatments after failure	Proportion of panellists agreed (%)
Enrolment in clinical trials	100%
Splenectomy	100%
Mycophenolate	89%
Rituximab (375 mg/m^2^ once a week for 4 weeks)	100%
Cyclophosphamide (1–2 mg/kg per day)	100%
Rituximab (1,000 mg given twice, 2 weeks apart)	90%

Third‐ or later‐line treatments after relapse	Proportion of panellists agreed (%)
Cyclophosphamide (1–2 mg/kg per day)	90%
Enrolment in clinical trials	89%
Splenectomy	100%
Mycophenolate	89%
Rituximab (375 mg/m^2^ once a week for 4 weeks)	89%

Treatment considerations for secondary wAIHA	Proportion of panellists agreed (%)
Treatment of underlying disease is the first choice	100%
Treatment of underlying diseases may lead to a remission of the AIHA	100%
Comorbidities	89%
Immunosuppressants are not lower priority	80%

First‐line therapy for patients with wAIHA secondary to CTD	Proportion of panellists agreed (%)
Corticosteroids	100%
Steroid and MMF	90%
Treat CTD first	90%

Later‐line therapy for patients with wAIHA secondary to CTD	Proportion of panellists agreed (%)
Rituximab	89%
Immunosuppressants	100%
CNI	90%
Steroid pulse	100%
Corticosteroid + rituximab	80%
MMF	100%

Preferred treatment options in later lines of therapy for patients with primary wAIHA	Proportion of panellists agreed (%)
Second line: standard immunosuppressants (AZA, IVIG or rituximab)	80%
Second line: IVIG; MMF, AZA, CNI	80%
Third line: rituximab	80%
Rituximab + steroids, or MMF	80%
Rituximab	90%
Anti‐CD20 antibodies	90%

Situations for considering blood transfusion for the management of wAIHA	Proportion of panellists agreed (%)
Hb <7 g/dL, or <9 g/dL if there is comorbid ischemic heart disease	89%
Severe symptomatic anaemia (Hb <5 g/dL)	90%
Severe anaemia (Hb <6 g/dL)	80%
Hb <7 g/dL and symptom	89%
Hb is <7 g/dL and moderate and severe anaemia symptoms	100%
Severe symptomatic anaemia, refractory to steroids	90%
Life‐threatening condition	100%
Planned surgery	90%
Severe complications	90%

The most relevant clinical indicators to ensure appropriate follow‐up and monitoring of patients with wAIHA	Proportion of panellists agreed (%)
Hb levels	89%
Haptoglobin and LDH: weekly monitoring if the patient has been admitted for other reasons, otherwise it could be every 1–3 months	80%
Hb level, reticulocyte count, haptoglobin	100%
Hb level: monitoring at least once every 3 months	80%
Hb, LDH and reticulocyte count: monitoring every 2 months if stable, otherwise, every month	90%

Situations in which to consider treatment switch or add‐on for patients with wAIHA	Proportion of panellists agreed (%)
Time to wait for a response depends on the drugs, e.g., corticosteroids expect rapid response every day, whereas rituximab we expect over months	80%
Relapse is defined as a drop in Hb to < 10 g/dL due to haemolysis	80%
Relapse is defined as an initial response and then deterioration of Hb supported by evidence of active haemolysis	90%
Recurrence is defined the same as relapse	100%
Complications	88%
Patient preference	88%

Signs of ‘lack of response’ in wAIHA	Proportion of panellists agreed (%)
Still transfusion dependent	89%
No parameter change (Hb, LDH, reticulocytes, etc.) in 4 weeks	90%
Hb levels, symptoms of anaemia	89%

Management of wAIHA comorbidities and potential complications (e.g., haemolysis‐related organ dysfunction or thrombotic events)	Proportion of panellists agreed (%)
Appropriate management of complications	89%
Minimise steroid toxicities	89%
Minimise steroid use and early combination with MMF or AZA	90%
Increase Hb to avoid heart failure, anti‐platelet medicine for patients who experienced thrombotic events such as brain infarction and ischemic heart disease	90%
Adverse effects of glucocorticoids and immunosuppressants	90%

Abbreviations: AIHA: autoimmune haemolytic anaemia; AZA: azathioprine; CNI: calcineurin inhibitor; CTD: connective tissue disease; Hb: haemoglobin; IVIG: intravenous immunoglobulin; LDH: lactate dehydrogenase; MMF: mycophenolate mofetil; wAIHA: warm autoimmune haemolytic anaemia.

**TABLE 4:** Consensus statements related to wAIHA management challenges and research priorities.

The current challenges to diagnose wAIHA	Proportion of panellists agreed (%)
Steroid complications	100%
Differential diagnosis in AIHA (wAIHA, CAD or mixed AIHA) is sometimes challenging, and Coombs‐negative wAIHA is often difficult to diagnose	90%
Low disease awareness of physicians and patients. As some wAIHA patients have low antibody titres, more sensitive diagnostic tests are needed	80%
Heterogeneity of disease manifestations	80%
Lack of standardised guidelines	100%

Warm AIHA treatment gaps that need to be addressed with currently available options, including off‐label treatments (e.g., immunosuppressants, immunoglobulins, rituximab, etc.)	Proportion of panellists agreed (%)
Rituximab is not approved	90%
Rituximab is expensive	80%
The absence of an evidence‐based algorithm due to lack of high‐quality clinical trials	100%

Challenges or barriers to overall management of wAIHA patients	Proportion of panellists agreed (%)
The absence of an evidence‐based algorithm due to lack of high‐quality clinical trials	89%
Treatments not being reimbursed	80%
The duration of immunosuppression maintenance is not clear and multiple team involvement can be confusing	100%
Refractory disease	100%
Unpredictable relapse and low awareness of mortality risk	100%
Lack of awareness about the side effects of PSL for physicians. Off‐label of rituximab and immunosuppressants, most haematologists believe Hb = 7 mg/dL is acceptable for wAIHA patients, although they are anaemic. Ninety percent are oncologists and under 10% is haematologists, so most of wAIHA patients see oncologists who do not have enough knowledge and experience	80%
Development of new therapies	100%

Further research and evidence‐generation approaches that should be considered to address the evidence gaps in the published literature for wAIHA, specifically in the APAC region	Proportion of panellists agreed (%)
Newer biological therapies to reduce the need to resort to splenectomy	100%
What causes poor prognosis in wAIHA in APAC? Information about comorbidities of wAIHA (thrombosis, malignancies etc.) is lacking	100%
New treatment option except steroid is very promising	100%
Incidence and outcomes in the post‐rituximab era	80%
A drug that has proven effectiveness through clinical trials is needed	89%
Medical advisor boards, Scientific meetings, comparing guidelines and real clinical practice in each country, registration of wAIHA, survey the national and health insurance database	100%
Clinical trials comparing corticosteroids with other agents in the first‐line setting	100%
Clinical trials comparing various second‐line options	100%
Assessment of quality of life for wAIHA patients	100%
Awareness about wAIHA among healthcare professionals	100%

Abbreviations: AIHA: autoimmune haemolytic anaemia; APAC: Asian‐Pacific region; CAD: cold agglutination disease; wAIHA: warm autoimmune haemolytic anaemia.

#### 3.2.1 Diagnosis and Screening Practices

Panellists agreed that key tests for wAIHA diagnosis, supplemental to DAT, include screening for underlying diseases (e.g., cancer, including chronic lymphocytic leukaemia [CLL], infections, drug/medication use or autoimmune disorders, including SLE), spherocytes and rheumatoid factor to exclude rheumatoid arthritis (Table 2).

Persistent lymphadenopathy (> 1 cm), splenomegaly, cytopenia or monoclonal gammopathy should prompt evaluation for lymphoproliferative disease.

#### 3.2.2 Interventions

Panellists agreed that intervention is warranted in cases with Hb < 10 mg/dL, severe anaemia symptoms or underlying wAIHA‐associated diseases (Table 3). Short‐term goals include stabilising Hb, improving anaemia symptoms and inducing remission. Long‐term goals span treatment cessation, quality of life improvement, symptom control, relapse prevention, reduced steroid burden and avoiding complications like infections or thrombosis.

Consensus was reached on the use of corticosteroids as first‐line wAIHA treatment, including 1 mg/kg prednisone daily for 3 weeks or 250–1000 mg IV methylprednisolone daily for 1–3 days. Panellists agreed that 40 mg dexamethasone for 4 days every 4 weeks should not be used as first‐line therapy. The most effective first‐line therapy in patients with wAIHA secondary to CTD is corticosteroids, steroids and MMF, while prioritising the treatment of the underlying CTD.

Second‐line treatments following corticosteroid failure are immunosuppressants, including cyclosporine at 2–5 mg/kg per day divided into two doses; AZA at 1–2 mg/kg/day divided into multiple doses; and rituximab at 375 mg/m^2^ once a week for 4 weeks or 1000 mg given twice, 2 weeks apart, with or without corticosteroids. Panellists agreed that second‐line treatments after relapse are the same as after failure, but enrolment in a clinical trial would be appropriate. Panellists agreed that third‐line options following treatment failure or relapse include splenectomy, immunosuppressants (MMF, cyclophosphamide and rituximab) and clinical trial enrolment. There was also agreement for the treatment options for later lines were rituximab, immunosuppressants, calcineurin inhibitors (CNIs), steroid pulses, a combination of corticosteroids and rituximab, and MMF. Panellists unanimously rejected the use of TNF‐α inhibitors in later lines.

#### 3.2.3 Follow‐Up and Monitoring

Panellists reached consensus on several clinical indicators that could be used to ensure appropriate follow‐up and monitoring of patients with wAIHA: Hb, haptoglobin and LDH levels, and reticulocyte count (Table 3). Panellists also agreed that blood transfusion should be considered for patients with Hb < 7 g/dL or < 9 g/dL with comorbid ischaemic heart disease, severe symptomatic anaemia (e.g. Hb < 5–6 g/dL), severe anaemia refractory to corticosteroids, life‐threatening conditions, planned surgeries or severe complications. The need for transfusion may also vary depending on age and comorbidities, such as chronic heart failure.

#### 3.2.4 Treatment Modifications

Clinicians should switch or enhance wAIHA therapy when patients do not respond, relapse, experience recurrence or complications, or would prefer to switch therapies (Table 3). Consensus indicators for a lack of response are transfusion dependence, no change in haematological parameters (Hb, LDH, reticulocyte count, etc.) over 4 weeks, and persistent symptoms of anaemia.

#### 3.2.5 Comorbidities and Complications

Panellists reached consensus on several key strategies for managing the primary concerns related to comorbidities and potential complications associated with wAIHA (Table 3). Attending physicians should manage all complications appropriately, minimise steroid toxicities, reduce steroid use and consider early combination therapy with MMF or AZA. Panellists agreed that increasing Hb levels is important for heart failure management. Anti‐platelet medications may be prescribed to patients with thrombotic events such as brain infarction or ischaemic heart disease. Furthermore, attention should be given to adverse effects from glucocorticoids and immunosuppressants.

#### 3.2.6 Diagnostic Challenges

Consensus was reached on current barriers to diagnosing patients with wAIHA, including low disease awareness of physicians and patients, heterogeneity of disease manifestations and lack of standardised guidelines (Table 4). While symptomatic anaemia is well‐recognised, differential diagnosis of AIHA subtypes, such as wAIHA, cold agglutinin disease (CAD) or mixed AIHA, can be challenging. DAT‐negative wAIHA is also often difficult to diagnose. Finally, more sensitive diagnostic tests are needed to diagnose wAIHA in those with low antibody titres.

#### 3.2.7 Unmet Needs

Panellists reached consensus on three wAIHA treatment gaps that need to be addressed with currently available medications, including off‐label treatments: (i) approval for rituximab across the region, (ii) improved affordability of rituximab and (iii) formalisation of a wAIHA treatment algorithm based on high‐quality clinical trials. It was noted that the use of rituximab as an earlier line of treatment might reduce relapse rates, with rituximab being the only effective biological treatment available, highlighting a need for improved access to this therapy.

Challenges in the overall management of patients with wAIHA include the perception that steroids are the only effective treatment option and a lack of awareness among physicians about the side effects of prednisone and other steroids. Panellists also noted additional challenges, including drug reimbursement, uncertainty of the duration of maintenance immunosuppression, the unpredictability of relapse and the involvement of multiple teams in patient management leading to confusion. Overall, panellists agreed on the need for new therapeutic options, due to patients continuing to experience chronic fatigue and anaemia leading to low quality of life.

#### 3.2.8 Research Priorities

Panellists agreed on the necessity for increasing awareness about wAIHA among healthcare professionals through the establishment of patient advocacy groups, medical advisory boards and scientific meetings. They also highlighted the importance of comparing guidelines with real‐world practice across countries, registering patients and utilising national insurance databases. Consensus was reached on key research priorities to address evidence gaps in the APAC region:

Optimisation of treatment strategies:

- Clinical trials comparing first‐ and second‐line therapies;
- Development of steroid‐sparing treatments and novel biologics to reduce reliance on splenectomy;

Long‐term outcomes and epidemiology:

- Studies on incidence and outcomes post rituximab;
- Research into poor prognostic factors, including comorbidities such as thrombosis and malignancy;

Patient‐centred outcomes

- Evaluating quality of life in patients with wAIHA.

## 4 Discussion

This study aimed to summarise the existing literature on wAIHA in the APAC region and to establish expert consensus on its diagnosis, treatment and management through a Delphi panel. The SLR identified 37 studies, most focusing on the clinical burden of AIHA and wAIHA, with limited and often outdated data on epidemiology and economic burden, suggesting that more population‐based studies are needed. Notably, no studies reported on quality of life, highlighting a significant gap in understanding the magnitude of the humanistic burden of wAIHA.

The SLR confirmed that wAIHA was the most common subtype of AIHA in the APAC region, with varying proportions of secondary AIHA. High mortality rates were reported, particularly among patients with secondary AIHA and those with underlying malignancies. Treatment guidelines from China, Japan and South Korea recommended corticosteroids as the first‐line treatment, with immunosuppressants and rituximab as second‐line options. Reported treatment patterns confirmed that these were the preferred treatments. However, the studies also showed that there were still differences in treatment for wAIHA, so there is a need for an evidence‐based prioritisation strategy that accounts for each patient's clinical status as well as the practical factors of drug availability, including approval status and reimbursement policy.

Though first‐line use of corticosteroids is initially effective at controlling wAIHA‐associated pathologies and is the approach recommended by clinical expert panellists, they are associated with severe side effects, including osteoporosis, diabetes, cardiovascular complications and increased susceptibility to infections [51, 52, 53]. Not only do these side effects reduce patient quality of life, they also contribute to overall morbidity and mortality [54]. Given these documented adverse effects, continued reliance on corticosteroids indicates an urgent need for new effective therapeutic options for wAIHA treatment.

Several established and novel agents are being investigated for their efficacy in treating wAIHA [55, 56, 57, 58]. Plasma cell‐targeting therapies that have recently undergone or are undergoing clinical trials in patients with wAIHA include parsaclisib, ibrutinib, rilzabrutnib, bortezomib, daratumumab, isatuximab, ianalumab, obexelimab and povetacicept. These agents could help control wAIHA by reducing the production of pathogenic autoantibodies. Beyond B‐cell–targeted therapies, the spleen tyrosine kinase (Syk) inhibitors fostamatinib and sovleplenib offer a strategy to disrupt signalling downstream of the Fc receptor, thereby limiting phagocytosis of antibody‐coated red blood cells. Similarly, blockade of the neonatal Fc receptor (FcRn) with nipocalimab, orilanolimab or batoclimab reduces recycling of pathogenic IgG autoantibodies, leading to decreased serum levels. Complement inhibition is also being actively explored, with agents such as the C3 inhibitor pegcetacoplan showing promise in preventing activation of the complement cascade and subsequent haemolysis. Collectively, these novel targeted therapies have the potential to shift the treatment paradigm in wAIHA by offering more precise, effective and safer alternatives to corticosteroids.

Rituximab has emerged as an effective second‐line treatment option for wAIHA, particularly in cases where corticosteroids fail or are not tolerated. Several studies included in the SLR demonstrated the efficacy of rituximab in improving clinical outcomes for patients with wAIHA. Despite its effectiveness, access to rituximab remains a challenge in some APAC countries due to high costs and a lack of approval. However, its use is associated with significant immunosuppressive effects, including an increased risk of infections, hypogammaglobulinemia and delayed immune reconstitution [59]. These concerns necessitate careful patient selection and monitoring when prescribing rituximab.

### 4.1 Strengths and Limitations

The strengths of the study include a comprehensive literature review that has identified key gaps in the existing research and an expert Delphi panel providing practical recommendations for wAIHA management. Although representatives from the most populous APAC regions were included, not all countries were represented, so the consensus statements might not fully apply to all countries based on differences in healthcare systems, diagnostic criteria and treatment availability.

### 4.2 Conclusion

This study highlights the need for evidence‐based, prioritised treatment guidelines that align with real‐world constraints. The SLR and Delphi panel have shown significant gaps in both the evidence base and current clinical practice, particularly concerning epidemiology, treatment outcomes and quality of life for patients with wAIHA. The resulting consensus recommendations provide a framework to improve diagnosis, treatment and overall disease management. Future research priorities include conducting large‐scale epidemiological studies, quality‐of‐life assessments, comparative clinical trials and the development of new treatment options to address these identified gaps and improve patient outcomes.

## Author Contributions

B.W., N.S. and P.T. conceived the study. R.B., P.K. and D.A. developed the methodology and analysed the results. P.K. drafted the manuscript. All authors critically reviewed drafts and approved the final manuscript. The experts listed as authors on this manuscript provided input and support throughout the development of the Delphi panel process and contributed to the interpretation and contextualisation of the findings.

## Funding

This study was sponsored and funded by Johnson & Johnson Innovative Medicine.

## Ethics Statement

This work used previously published materials and did not involve human or animal participants; therefore, no ethics approval was required.

## Conflicts of Interest

B.W., N.S. and P.T. are employees of Johnson & Johnson Innovative Medicine. P.K., R.B. and D.A. are employees of Amaris Consulting, which received funding from Johnson & Johnson Innovative Medicine for the study.

## Supporting information




**Table S1**: Search terms for EMBASE and MEDLINE via www.embase.com on 12 October 2022. **Table S2**: Search terms for Wanfang via www.wanfangdata.com.cn on 12 October 2022. **Table S3**: Search terms for CNKI via www.kns.cnki.net on 12 October 2022. **Table S4**: Search terms for KoreaMed via www.koreamed.org on 12 October 2022. **Table S5**: Search terms for Ichushi via www.jamas.or.jp on 12 October 2022. **Table S6**: List of conference proceedings. **Table S7**: List of excluded citations. **Table S8**: Characteristics of included studies (n = 37). **Table S8**: Characteristics of included studies (n = 37). **Table S9**: Statements not reaching consensus. **Table S10**: Statements with consensus disagreement. **Table S11**: Summary of Delphi survey responses of part 1.1 Q1: what are the most important supplementary tests to consider during screening of patients, in addition to main test (direct antiglobulin test [DAT or Coombs test] to detect IgG + C3d or IgG) in current clinical guidelines? **Table S12**: Summary of Delphi survey responses of part 1.1 Q2: what are the optimal strategies for evaluating and addressing the underlying diseases that cause secondary wAIHA? **Table S13**: Summary of Delphi survey responses of part 1.2 Q1: how you determine when intervention is needed versus “watch and wait” (i.e. what factors should be considered for treatment initiation in wAIHA)? **Table S14**: Summary of Delphi survey responses of part 1.2 Q2: what are some of the preferrable treatment options in later lines (e.g., second line, third line, or after steroids) of therapy in patients with primary wAIHA? (Please specify dosing, frequency, and duration for each treatment)? **Table S15**: Summary of Delphi survey responses of part 1.2 Q3: what are the short‐term and long‐term treatment goals in patients with wAIHA? **Table S16**: Summary of Delphi survey responses of part 1.2 Q4: what are the most relevant clinical indicators that could be used to ensure appropriate follow‐up and monitoring of patients with wAIHA? **Table S17**: Summary of Delphi survey responses of part 1.2 Q5: in what cases should blood transfusion be considered for management in wAIHA, considering the potential challenges associated with alloimmunisation and delay of haemolytic reaction? **Table S18**: Summary of Delphi survey responses of part 1.2 Q6: treatment options and preferences in wAIHA per treatment line and after failure or relapse. **Table S19**: Summary of Delphi survey responses of part 1.2 Q7: regarding will the treatment options be affected by the underlying disease in cases of secondary wAIHA? What are the most effective treatment options in first line of therapy in patients with wAIHA secondary to connective tissue diseases (CTD)? What are the most effective treatment options in later lines of therapy in patients with wAIHA secondary to connective tissue diseases (CTD)? **Table S20**: Summary of Delphi survey responses of part 1.2 Q8: when to consider treatment switch or add‐on for patients with wAIHA? **Table S21**: Summary of Delphi survey responses of part 1.2 Q9: how do you manage the primary concerns in the management of comorbidities and potential complications associated with wAIHA, such as haemolysis‐related organ dysfunction or thrombotic events? **Table S22**: Summary of Delphi survey responses of part 2 Q1: what are the current challenges to diagnose wAIHA patients? **Table S23**: Summary of Delphi survey responses of part 2 Q2: what are the current gaps in the treatment of wAIHA that need to be addressed with current available options, including off‐label treatments (e.g., immunosuppressants, immunoglobulins, rituximab, etc.) in your country? **Table S24**: Summary of Delphi survey responses of part 2 Q3: what are the current challenges in or barriers to overall management of wAIHA patients? **Table S25**: Summary of Delphi survey responses of part 2 Q4: what potential further research and evidence generation approaches should be considered to address the evidence gaps in the published literature for wAIHA, specifically in the APAC region?

## Data Availability

All data included in the SLR section of this study are publicly available from the original publications from which data were extracted. The original contributions presented in the study are included within the article and its Supporting Information. Further inquiries can be directed to the corresponding author.
