# Supplementary material for: Warm Autoimmune Haemolytic Anaemia Management in Asia‐Pacific: A Delphi Panel Expert Consensus and Systematic Literature Review
Source: EJHaem. 2025 Nov 6;6(6):e70174. doi: 10.1002/jha2.70174 (PMC12591179; doi:10.1002/jha2.70174)
Supplement: Supplementary file 1 — Table S1: Search terms for EMBASE and MEDLINE via www.embase.com on 12 October 2022. Table S2: Search terms for Wanfang via www.wanfangdata.com.cn on 12 October 2022. Table S3: Search terms for CNKI via www.kns.cnki.net on 12 October 2022. Table S4: Search terms for KoreaMed via www.koreamed.org on 12 October 2022. Table S5: Search terms for Ichushi via www.jamas.or.jp on 12 October 2022. Table S6: List of conference proceedings. Table S7: List of excluded citations. Table S8: Characteristics of included studies (n = 37). Table S8: Characteristics of included studies (n = 37). Table S9: Statements not reaching consensus. Table S10: Statements with consensus disagreement. Table S11: Summary of Delphi survey responses of part 1.1 Q1: what are the most important supplementary tests to consider during screening of patients, in addition to main test (direct antiglobulin test [DAT or Coombs test] to detect IgG + C3d or IgG) in current clinical guidelines? Table S12: Summary of Delphi survey responses of part 1.1 Q2: what are the optimal strategies for evaluating and addressing the underlying diseases that cause secondary wAIHA? Table S13: Summary of Delphi survey responses of part 1.2 Q1: how you determine when intervention is needed versus “watch and wait” (i.e. what factors should be considered for treatment initiation in wAIHA)? Table S14: Summary of Delphi survey responses of part 1.2 Q2: what are some of the preferrable treatment options in later lines (e.g., second line, third line, or after steroids) of therapy in patients with primary wAIHA? (Please specify dosing, frequency, and duration for each treatment)? Table S15: Summary of Delphi survey responses of part 1.2 Q3: what are the short‐term and long‐term treatment goals in patients with wAIHA? Table S16: Summary of Delphi survey responses of part 1.2 Q4: what are the most relevant clinical indicators that could be used to ensure appropriate follow‐up and monitoring of patients with wAIHA? Table S17: Summary of [file JHA2-6-e70174-s001.pdf]

Supplemental Materials

1.1 Appendix S1

Table S1. Search terms for EMBASE and MEDLINE via www.embase.com on 12 October 2022

No	Query	Results
#1	waiha OR 'warm autoimmune hemolytic anemia'/exp OR 'warm autoimmune hemolytic anemia'/de OR 'warm antibody hemolytic anemia' OR 'autoimmune hemolytic anemia'/exp OR 'autoimmune hemolytic anemia'/de OR 'antibody hemolytic anemia' OR 'primary warm autoimmune hemolytic anemia' OR 'secondary warm autoimmune hemolytic anemia'	12,002
#2	china:ti,ab,ad,ff OR india:ti,ab,ad,ff OR japan:ti,ab,ad,ff OR 'republic of korea':ti,ab,ad,ff OR australia:ti,ab,ad,ff OR taiwan:ti,ab,ad,ff OR asia*:ti,ab OR korea:ti,ab,ad,ff OR philippines:ti,ab,ad,ff OR 'singapore':ti,ab,ad,ff OR 'malaysia':ti,ab,ad,ff OR 'hong kong':ti,ab,ad,ff OR 'new zealand':ti,ab,ad,ff OR thailand:ti,ab,ad,ff OR vietnam:ti,ab,ad,ff	8,583,648
#3	#1 ANerrD #2	2,028
#4	epidemiology:ti,ab OR incidence:ti,ab OR inciden*:ti,ab OR prevalence:ti,ab OR prevalen*:ti,ab OR morbidity:ti,ab OR mortality:ti,ab OR 'risk factor*':ti,ab OR 'transfus*':ti,ab OR 'autoantibody'/de OR 'anti-red blood cell autoantibod*' OR 'splenectomy'/de OR 'steroid'/de OR 'steroid tapering' OR 'refractory anemia'/de OR (‘rituximab’ near/3 ‘refractory’) or immunosuppressant	4,979,275
#5	'socioeconomics'/de OR 'cost benefit analysis'/de OR 'cost of illness'/de OR 'cost control'/de OR 'economic aspect'/de OR 'health care cost'/de OR 'health care financing'/de OR 'health economics'/de OR 'hospital cost'/de OR fiscal:ti OR financial:ti OR finance:ti OR funding:ti OR 'cost minimization analysis'/exp OR ((cost NEAR/1 estimate$):ti,ab) OR ((cost NEAR/1 variable$):ti,ab) OR ((unit NEAR/1 cost$):ti,ab) OR cost:ti OR 'productivity loss'/de OR ((work NEAR/3 productivity):ti,ab)	765,056
#6	'quality adjusted life year'/de OR 'quality of life index'/de OR 'sickness impact profile'/de OR ((quality NEAR/2 (wellbeing OR 'well being')):ti,ab) OR qal*:ti,ab OR qtime*:ti,ab OR qwb*:ti,ab OR daly*:ti,ab OR qol*:ti,ab OR hql*:ti,ab OR hqol*:ti,ab OR hrqol*:ti,ab OR 'eq 5d':ti,ab OR 'sf 36':ti,ab OR 'whoqol bref':ti,ab OR 'visual analog scale':ti,ab	244,426
#7	#4 OR #5 OR #6	5,761,409
#8	#3 AND #7	903
#9	#8 AND ('article'/it OR 'article in press'/it)	562

Table S2. Search terms for Wanfang via www.wanfangdata.com.cn on 12 October 2022

No	Query	Results
#1	主题:("自身免疫性溶血性贫血") or 题名或关键词:("自身免疫性溶血性贫血") or 主题:("温抗体型自身免疫性溶血性贫血") or 题名或关键词:("温抗体型自身免疫性溶血性贫血")	1,687
#2	主题:(发病率 or 患病率 or 死亡率 or 流行病* or 流行病研究 or 危险因素 or 风险因素 or 输血 or 红细胞输注 or 输血负担 or 自身抗体 or 抗红细胞抗体 or 脾切除 or 类固醇减量 or 糖皮质激素减量 or 难治性贫血) or 主题:(利妥昔单抗/NEAR 3 无效)	1,272,039
#3	主题:(费用 or 花费 or 经济负担 or 治疗费用 or 疾病花费)	465,742
#4	(主题:(疾病负担 or 临床负担 or 生产力损失 or 劳动力损失 or 工作损失) or 题名或关键词:(临床/NEAR 2 负担)) or (主题:(生命质量评价 or 生命质量* or 生产力 or 劳动力 or 工作) or 题名或关键词:(患者/NEAR 2 负担))	8,832,794
#5	#2 OR #3 OR #4	10,331,937
#6	#1 AND #5	1,005
#7	Limit #6 to 2010-2022	632

Table S3. Search terms for CNKI via www.kns.cnki.net on 12 October 2022

No	Query	Results
#1	SU='自身免疫性溶血性贫血' OR SU='温抗体型自身免疫性溶血性贫血'	1,180
#2	TKA='自身免疫性溶血性贫血' OR TKA='温抗体型自身免疫性溶血性贫血'	1,759
#3	TKA=('发病率'+'患病率'+'死亡率'+'流行病学'+'流行病研究'+'危险因素'+'风险因素'+'输血'+'红细胞输注'+'输血负担'+'自身抗体'+'抗红细胞抗体'+'脾切除'+'类固醇减量'+'糖皮质激素减量'+'难治性贫血'+'利妥昔单抗/NEAR 3 无效')	993,979
#4	TKA=('费用'+'花费'+'经济负担'+'治疗费用'+'疾病花费')	384,446
#5	TKA=('疾病负担'+'临床负担'+'生产力损失'+'工作损失'+'劳动力损失'+'生产力'+'劳动力'+'工作'+'临床/NEAR 2 负担'+'生命质量评价'+'生活质量'+'患者/NEAR 2 负担')	7,036,591
#6	#1 OR #2	1,760
#7	#3 OR #4 OR #5	8,231,497
#8	#6 AND #7	697
#9	Limit#8 to 2010-2022	434

Table S4. Search terms for KoreaMed via www.koreamed.org on 12 October 2022

No	Query	Results
#1	wAIHA[ALL] OR (warm[ALL] AND autoimmune[ALL] AND hemolytic[ALL] AND anemia[ALL]) OR 와이하[ALL] AND (온열[ALL] AND 자가면역[ALL] AND 용혈성[ALL] AND 빈혈[ALL])	5

Table S5. Search terms for Ichushi via www.jamas.or.jp on 12 October 2022

No	Query	Results
#1	温式自己免疫性溶血性貧血/AL	56
#2	(#1) and ((FT=Y))	28

Table S6. List of conference proceedings

Conference Proceedings
Blood conference<br>Chinese Society of Hematology<br>Chinese Alliance of Society for Hematology<br>Japanese Society of Hematology<br>The Korean Society of Hematology<br>Taiwan Society of Blood and Marrow Transplantation<br>India Society of hematology and blood transfusion<br>Philippine Society of hematology and transfusion medicine<br>Singapore society of hematology<br>Malaysian society of hematology

Table S7. List of excluded citations

No.	Reference	Reason for exclusion
1	Kashyap, R., Singh, A., Kumar, P; (2016). Prevalence of autoimmune hemolytic anemia in multiple myeloma: A prospective study. Asia-Pacific Journal of Clinical Oncology. 12. 10.1111/ajco.12230	Not population of interest
2	Venkatachala, R.P., Sheela, C.N., Anandram, S., Ross, C.R. (2021). Autoimmune Hemolytic Anemias in Pregnancy: Experience in a Tertiary Care Hospital in South India. Journal of Obstetrics and Gynecology of India. 71. 10.1007/s13224-021-01443-8	Not population of interest
3	Das, S.S., Nityanand, S., Chaudhary, R. (2009). Clinical and serological characterization of autoimmune hemolytic anemia in a tertiary care hospital in North India. Annals of Hematology. 88. 10.1007/s00277-008-0674-6	Not outcomes of interest
4	Higuchi, T., Hoshi, T., Toriyama, M., Nakajima, A., Haruki, K. (2022). Infrequent Thrombotic Complications in Japanese Patients with Warm Autoimmune Hemolytic Anemia. Internal medicine (Tokyo, Japan). 10.2169/internalmedicine.0541-22	Not outcomes of interest
5	Zhang, L.-N., Liu, X.-F., Li, Y. (2019). Serological Characteristics of Patients with Autoimmune Hemoly-tic Anemia and Efficacy and Safety of Incompatible Transfusion of Red Blood Cells. Zhongguo shi yan xue ye xue za zhi. 27. 10.19746/j.cnki.issn.1009-2137.2019.03.045	Not outcomes of interest
6	Choudhry, V.P., Passi, G.R., Pati, H.P. (1996). Clinico-hematological spectrum of auto-immune hemolytic anemia: an Indian experience. The Journal of the Association of Physicians of India	Not outcomes of interest
7	Yang, N., Wang, B., Gao, F., Huang, X.-Y., Zhao, X.-L., Wang, Y.-Y., Zhang, X.-P. (2020). Evaluation of Efficacy and Safety of Blood Transfusion and Hormone Therapy in 40 Patients with Autoimmune Hemolytic Anemia. Zhongguo shi yan xue ye xue za zhi. 28. 10.19746/j.cnki.issn.1009-2137.2020.04.038	Not outcomes of interest
8	Zhou, J.-C., Wu, M.-Q., Peng, Z.-M., Zhao, W.-H., Bai, Z.-J., Bush, E. (2020). Clinical analysis of 20 patients with non-Hodgkin lymphoma and autoimmune hemolytic anemia: A retrospective study. Medicine (United States). 99. 10.1097/MD.0000000000019015	Not outcomes of interest
9	Xing, L.M., Shao, Z.H., Liu, H., Shi, J., Bai, J., Wang, H.Q., Tu, M.F., Sun, J. (2006). The clinical features of the autoimmune hemolytic anemia with both warm and cold autoantibodies. Zhonghua xue ye xue za zhi = Zhonghua xueyexue zazhi. 27	Not outcomes of interest

10	Liu, H., Li, W., Gao, Q., Jiang, D. (2020). Systematic analysis of cases of lymphoma with autoimmune hemocytopenia reported in the Chinese literature in the past 30 years. 10.3969/j.issn.1000-8179.2020.02.032	Not population of interest
11	Sun, D.-P., Wang, L., Zhang, R., Xu, J., Tian, T., Fan, L., Xu, W., Li, J.-Y. (2015). Clinical Analysis of Anemia in Castleman Disease. 10.7534/j.issn.1009-2137.2015.03.019	Not outcomes of interest
12	Feng, Y.Y., Xing, L.M. (2020). Progress in research on autoimmune hemolytic anemia secondary to lymphoproliferative diseases	Not outcomes of interest
13	Shi, J., Huan, X., Zhou, L., Xi, J., Song, J., Wang, Y., Luo, S., Zhao, C. (2021). Comorbid Autoimmune Diseases in Patients With Myasthenia Gravis: A Retrospective Cross-Sectional Study of a Chinese Cohort	Not outcomes of interest
14	Das, S.S., Chaudhary, R. (2009). Utility of adsorption techniques in serological evaluation of warm autoimmune hemolytic anemia	Not outcomes of interest
15	Liu, J., Li, M., Liang, J.-Y., Xu, X.-F., Zeng, W.-G., Wang, Y.-J., Jiang, Y., etal. (2021). Autoimmune diseases in HIV-negative cryptococcal meningitis	Not population of interest
16	王静, 于帆, 冯玉婷, 戴铭鑫, 张洪为, 黄远帅. (2022). 重度自身免疫性溶血性贫血患者临床特征及输注不同类型红细胞成分的疗效分析	Not outcomes of interest
17	邱启东, 阙庆和, 钟昌瑞, 陈维媚. (2022). RhCcEe 配合性血液输注对温抗体型 AIHA 患者输血安全性的影响	Not outcomes of interest
18	自身免疫性溶血性贫血诊疗指南(2022 年版)	Not outcomes of interest
19	董敏, 沈观樵. (2022). CD4+CD69+T 细胞在自身免疫性溶血性贫血患者外周血中的表达及临床意义	Not outcomes of interest
20	张联博, 李欣, 田勇. (2022). 自身免疫性溶血性贫血输血治疗方案回顾性分析	Not outcomes of interest
21	葛东梅, 张少强, 杨元铭, 刘姣, 鞠强, 王海燕. (2022). TPE 联合药物治疗低体重儿童慢性难治性自身免疫性溶血性贫血 1 例	Not outcomes of interest
22	刘向华. (2022). 不同输血方案在极重度自身免疫性溶血性贫血患者中的应用效果比较	Not population of interest
23	王莹. (2021). 血浆置换与去白细胞红细胞悬液输血在自身免疫性溶血性贫血中的应用	Not population of interest
24	邹丽萍. (2021). 血型基因检测在自身免疫性溶血性贫血患者输血治疗中的应用研究	Not outcomes of interest
25	钱姣, 徐苏娟. (2021). 自身免疫性溶血性贫血患者的输血方法选择和效果评价	Not outcomes of interest

26	赵凤莲, 马砚敏, 支君, 董予新. (2021). 自身免疫性溶血性贫血患者输血治疗效果观察	Not outcomes of interest
27	陈蕾, 邢莉民. (2021). 复发/难治性自身免疫性溶血性贫血的新药治疗现状	Not outcomes of interest
28	宋小彦. (2021). 探究不同输血方法用于自身免疫性溶血性贫血患者的临床疗效分析	Not outcomes of interest
29	关晶, 杨瑾, 邢莉民. (2021). CD20 单克隆抗体治疗自身免疫性溶血性贫血的进展	Not outcomes of interest
30	旷开其, 刘思圆, 张宁洁, 卜艳红, 桂嵘, 晏永和. (2021). 温自身抗体干扰抗体鉴定的应对策略	Not outcomes of interest
31	邸艳辉, 管行, 寇立舵, 白薇, 王新华. (2021). AIHA 患者血型血清学特征及输注悬浮红细胞疗效分析	Not outcomes of interest
32	张得胜, 王伟, 涂汉坤. (2021). 多种红细胞抗原相合性输血在自身免疫性溶血性贫血患者中的应用效果	Not outcomes of interest
33	张得胜, 王伟, 涂汉坤. (2021). 慢性肝病合并自身免疫性血液系统疾病的临床特征及应用糖皮质激素治疗效果分析	Not outcomes of interest
34	刘力铭. (2021). 输血科 152 例自身免疫性溶血性贫血患者不规则抗体筛查结果分析	Not outcomes of interest
35	段秉政, 连俊慧, 王淑莲, 陶琳, 高玉云. (2021). 直接抗人球蛋白试验抗体凝集强度的影响因素与临床意义	Not outcomes of interest
36	王中正. (2021). 自身抗体类型与输注红细胞制剂对自身免疫性溶血性贫血患者输血疗效的影响	Not population of interest
37	王雨涵, 王华, 王洁, 江灵, 黄远帅. (2021). RhCcEe 抗原配合性悬浮红细胞输注在温抗体型 AIHA 患者紧急输血中的应用	Not outcomes of interest
38	李森, 蓝夏璐, 蔡华聪, 朱铁楠, 王书杰, 赵永强. (2021). 伴血小板减少原发性抗磷脂综合征的临床特征及相关因素分析	Not outcomes of interest
39	陈新春, 邓绍团, 龙韵洪, 卢春生. (2020). 自身免疫性溶血性贫血抗体特征与不同输血方案疗效评估	Not population of interest
40	周红霞. (2020). 自身免疫性溶血性贫血患者的血型血清学特征及红细胞不相容输注的疗效及安全性研究	Not study type of interest
41	李森. (2020). 自身免疫性溶血性贫血对输血前血型鉴定、抗体筛查及交叉配血的作用分析	Not study type of interest
42	李明, 邓卫. (2020). 不同红细胞输注方式在自身免疫性溶血性贫血患者中的应用效果观察	Not outcomes of interest

43	杨月星. (2020). 自身免疫抗体检测在恶性血液疾病患者中的临床意义	Not outcomes of interest
44	党创伟, 周峰, 丁帅. (2020). 输注洗涤红细胞在临床中的应用效果研究	Not outcomes of interest
45	陈俊敢, 徐伟帆, 刘依宁. (2020). 血清稀释法在 1 例免疫性溶血性贫血患者输血中的应用	Not outcomes of interest
46	孙朝侠, 李伟望, 王志军, 施均, 毛强, 童春帆, 翟伟涛. (2020). 236 例血液病脾切除术围手术期并发症危险因素分析	Not outcomes of interest
47	梅丽春, 苏向珠, 朱亭西, 赵启波, 翟建昭, 武永康. (2020). 系统性红斑狼疮患者贫血特点分析	Not outcomes of interest
48	马利, 石静云, 吴涛, 汉英, 贾占武, 郭娟娟, 王存邦, 白海. (2019). 自身免疫性溶血性贫血合并急性造血功能停滞抢救成功一例并文献复习	Not outcomes of interest
49	洪梅. (2019). 自身免疫性溶血性贫血的诊疗现状	Not outcomes of interest
50	胡志平, 王东, 朱继业. (2019). 脾切除术在自身免疫性血液系统疾病治疗中的价值	Not outcomes of interest
51	李毅. (2019). 自身免疫性溶血性贫血患者输血方法的选择及效果分析	Not outcomes of interest
52	陈岚. (2019). 采用利妥昔单抗联合输血治疗自身免疫性溶血性贫血的观察	Not outcomes of interest
53	尹惠丽, 高凤岩. (2019). 不同红细胞制品治疗原发性 AIHA 的回顾性分析	Not outcomes of interest
54	周建明, 曹志林. (2019).直接抗人球蛋白试验阳性患者血型鉴定及交叉配血试验分析	Not outcomes of interest
55	肖艳丽, 赵一贺, 李代红. (2018). 直接 Coombs 试验阳性的 AIHA 患者的临床分析和输血治疗	Not outcomes of interest
56	黎海江, 冯学冠, 符晓玲, 杨艺伟, 黄植. (2018). 自身免疫性溶血性贫血对血型鉴定及交叉配血结果的影响	Not outcomes of interest
57	杨鹃, 乐爱平. (2018). 自身抗体和红细胞输注方式对自身免疫性溶血性贫血患者输血疗效影响	Not outcomes of interest
58	孔亚红, 滕景芬, 董文逸, 彭智鹏, 卢祥婵, 吴念宁. (2018). 140 例需输血 AIDS 病人自身抗体免疫类型与贫血相关性	Not outcomes of interest
59	王晓丽. (2018). 微柱凝集法与体外溶血不同配血方法在自身免疫性溶血性贫血患者中的应用价值分析	Not outcomes of interest
60	鲍瑞玲, 王闪. (2018). 自身免疫性溶血性贫血患者血型鉴定、抗体筛查及交叉配血的检测方法及结果分析	Not outcomes of interest

61	赖锦锋, 罗朝霞, 雷承泳. (2018). 自身免疫性溶血性贫血患者的输血疗效和血型血清学特征分析	Not outcomes of interest
62	高清妍, 刘晨曦, 李园, 彭广新, 李建平, 李洋, 叶蕾, 樊慧慧, 宋琳, 张莉, 井丽萍, 周康, 赵馨, 杨文睿, 武志洁, 杨洋, 熊佑祯, 张凤奎. (2017). 小剂量利妥昔单抗治疗 12 例难治/复发原发性自身免疫性溶血性贫血患者疗效分析	Not study type of interest
63	张妍杰. (2017). 自身免疫性溶血性贫血输血前检查的研究	Not study type of interest
64	牛天林. (2017). 自身免疫性溶血性贫血输血前的检查分析	Not study type of interest
65	夏荣, 张琦, 陈勤奋. (2017). 自身免疫性溶血性贫血患者输血前试验及临床输血专家共识	Not study type of interest
66	李玖平, 陈利红, 杨浩莹. (2017). 自身免疫性溶血性贫血对输血前血型鉴定、抗体筛查及交叉配血的影响	Not study type of interest
67	马会敏, 燕备战, 刘景汉, 兰炯采. (2017). 紧急情况红细胞 ABO 不同型输注及效果分析	Not study type of interest
68	王化泉, 何广胜, 李莉娟. (2017). 自身免疫性溶血性贫血诊断与治疗中国专家共识(2017 年版)	Not study type of interest
69	王立新, 陈春霞, 魏曾珍, 谭金哲, 吴秋月, 秦莉. (2017). 自身免疫性溶血性贫血患者输血治疗的回顾性分析	Not outcomes of interest
70	林俊华. (2016). 自身免疫性溶血性贫血患者输血前试验的应用价值	Not outcomes of interest
71	邵宗鸿, 郑萌颖. (2016). 自身免疫性溶血性贫血治疗进展	Not outcomes of interest
72	姚丽, 王忱. (2016). 温自身抗体的相关输血对策	Not outcomes of interest
73	张秋会, 胡兴斌, 孙文利, 安群星, 张玲玲, 张婧, 尹文. (2016). 免疫性疾病患者输血前血型血清学特征分析	Not outcomes of interest
74	韩芳. (2016). 溶血性贫血自身免疫型输血疗效与血清学特征及血型评估的临床意义	Not outcomes of interest
75	孙俊波, 陆应玉. (2016). 某三甲医院 2014 年临床应用洗涤红细胞的调查分析	Not outcomes of interest
76	徐泉元. (2016). 自身免疫性溶血性贫血患者成分输血的临床分析	Not outcomes of interest
77	李英梅, 孙慧. (2015). 自身免疫性溶血性贫血的诊断和治疗现状	Not outcomes of interest
78	马永华. (2015). 自身免疫性溶血性贫血血型血清学特征及输血治疗效果分析	Not outcomes of interest
79	陆欣, 王海军, 赵丽云, 李玉伟, 王助衡. (2015). 小剂量利妥昔单抗联合短期口服泼尼松治疗自身免疫性溶血性贫血的疗效	Not outcomes of interest

80	李志强. (2014). 诠释温抗体型 AIHA 血型血清学检测路径分析与输血原则	Not outcomes of interest
81	吴俣, 徐娟. (2014). 溶血性贫血治疗进展	Not outcomes of interest
82	梅玉霞, 庄承, 李远光, 刘小敏, 傅伟. (2014). 温抗体型自身免疫性溶血性贫血的诊治进展	Not outcomes of interest
83	孙小纯, 欧兴义, 林伟强, 陈亚军. (2014). 自身免疫性溶血性贫血患者输血前检查的研究	Not outcomes of interest
84	李雪英, 唐玉清, 张国平, 张华. (2014). 温抗体型自身免疫性溶血性贫血患者的血清学检测及输血对策	Not outcomes of interest
85	何鸣镝, 许婷婷, 沈钢. (2013). A_1 型患者检测出自身抗-A_11 例	Not outcomes of interest
86	于洋, 孙晓琳, 马春娅, 关晓珍, 张晓娟, 陈麟凤, et al. (2013). 61 例自身免疫性溶血性贫血患者血型血清学特征及输血疗效评估	Not outcomes of interest
87	张磊, 张烨, 郭楠, 刘亚庆, 刘素芳, 庄光艳, 范道旺. (2013). 自身免疫性溶血性贫血输血前检查的研究	Not outcomes of interest
88	贾晓伟. (2013). 55 例自身免疫性溶血性贫血实验诊断结果探讨	Not outcomes of interest
89	马晓璐, 魏莉. (2013). 1 名自身免疫性溶血性贫血患者的输血及护理	Not study type of interest
90	田进, 刘正华. (2013). 成人原发性自身免疫性溶血性贫血的治疗	Not study type of interesr
91	王芳, 毛伟, 李小红, 黄霞, 谭茜茜, 程磊. (2013). WARM 试剂在温性自身免疫性溶血性贫血中的应用	Not study type of interesr
92	孔祥骞. (2012). 自身免疫性溶血性贫血的实验室检测与临床输血疗效分析	Not study type of interesr
93	贾翠英, 单微, 代云峰. (2012). 自身免疫性溶血性贫血患者 ABO 正反定型不符 14 例	Not study type of interesr
94	刘代红. (2012). 药物诱发的免疫性溶血性贫血	Not study type of interesr
95	兰炯采. (2012). 加强对自身免疫性溶血性贫血输血前试验的研究	Not study type of interesr
96	孔庆芳. (2010). 贫血患者的免疫血液学检查结果分析	Not study type of interesr
97	亀崎, 豊実. (2013).【貧血　実臨床に役立つ診療のポイントと最新の知見】貧血の治療のポイント　温式自己免疫性溶血性貧血	Not study type of interesr
98	近江, 俊徳, 梶井, 英治, 小山田, 隆, 他. (1992). 温式自己免疫性溶血性貧血患者における赤血球結合 IgG 量の測定とその意義	Duplicate
99	曾月婷, 莫水群, 蔡肇丽, 梁结玲. (2022). 温抗体型自身免疫性溶血性贫血患者的输血策略探讨	Duplicate

100	中华人民共和国国家卫生健康委员会. (2022). 自身免疫性溶血性贫血诊疗指南(2022 年版)	Not population of interest
101	陈苗, 庄俊玲. (2017). 低剂量利妥昔单抗治疗温抗体型自身免疫性溶血性贫血的系统评价	Not population of interest
102	高钧明, 阚子凤, 赵芳, 闫蓓. (2017). 利妥昔单抗治疗温抗体型自身免疫性溶血性贫血的疗效评价	Not population of interest
103	孔秋红, 周凡, 刘景华, 刘彦琴, 王吉刚, 白颖, 郭步云, 吴丹彤. (2014). 低剂量利妥昔单抗治疗 15 例温抗体型自身免疫性溶血性贫血的疗效观察	Not population of interest
104	陆欣, 王海军, 赵丽云, 李玉伟, 王助衡. (2015). 利妥昔单抗治疗温抗体自身免疫性溶血性贫血的疗效评价	Not outcomes of interest
105	王吉刚, 周凡, 刘彦琴, 白颖, 刘景华, 张海婷, 李敏燕. (2015). 小剂量利妥昔单抗治疗难治及复发性温抗体型 AIHA 临床研究	Not outcomes of interest
106	陈新莲. (2018). 溶血性贫血患者的健康教育及护理干预措施	Not outcomes of interest
107	黄振东, 施均, 邵英起, 聂能, 张静, 李星鑫, 葛美丽, 黄金波, 郑以州. (2016). 12 例伴单克隆免疫球蛋白 Mκ 血症的自身免疫性溶血性贫血患者临床分析	Not outcomes of interest
108	甘东辉. (2014). 自身免疫性溶血性贫血的临床诊断	Not outcomes of interest
109	左雅蓓, 王艳, 林凤茹. (2012). 自身免疫性溶血性贫血诊治进展	Not outcomes of interest
110	陈瑶, 史小安. (2012). 75 例自身免疫性溶血性贫血免疫分型及临床分析	Not outcomes of interest
111	金皎. (2010). 自身免疫性溶血性贫血研究进展	Not outcomes of interest
112	车进, 张燕华, 麻静敏, 李凤, 陈兰兰, 李天君, 李美霖. (2015). 献血者抗-Wra 的血清学研究——附 1 例报告	Not study type of interest
113	邱迟娥, 魏顺会, 殷应勇. (2016). 免疫性溶血性贫血与强制性脊柱炎	Not outcomes of interest
114	王妮. (2013). 自身免疫性溶血性贫血症状及其护理要点	Not outcomes of interest

Table S8. Characteristics of included studies (n=37)

Author (Year)	Study type	Database/centre (study period)	Population (Sample size: AIHA/wAIHA)	Outcomes Epidemiology	Clinical burden	Economic burden
China						
Zhang (2021)[20]	Retrospective	Luohe Central Hospital (2018-2020)	AIHA (122/99)	✓		
Zhou (2021)[15]	Retrospective	Not reported	AIHA (NR/NR)	✓		
Huang (2014)[13]	Retrospective	Not reported	AIHA (NR/NR)	✓		
Wen (2015)[26]	Cross-sectional	Not reported	AIHA (NR/NR)	✓		
Chen (2020)[16]	Retrospective	West China Hospital (2009–2015)	AIHA (450/438)	✓	✓	
Fan (2019)[58]	Retrospective	Hematology Hospital of Chinese Academy (1996–2017)	Relapsed/refractory AIHA (30/13)		✓	
Lv (2019)[38]	Retrospective	Nanfang Hospital, Xiangya Hospital (2011–2016)	First allo-HSCT (26/NR)	✓	✓	
Xing (2019)[34]	Retrospective	Tianjin Medical University General Hospital (2012–2016)	AIHA (75/NR)		✓	
Sun (2018)[29]	Retrospective	Henan Third People's Hospital (2012–2016)	AIHA (60/NR)		✓	
Xiao (2018)[27]	Prospective	Tianjin first central hospital (2015–2018)	AIHA (88/78)		✓	
Zhang (2018)[59]	Retrospective	Hospital (2007–2017)	Patients with β-thalassemia who developed AIHA (5/NR)		✓	
Jin (2017)[40]	Prospective	Lishui People's Hospital (2012–2015)	AIHA (44/37)		✓	
Fang (2016)[43]	Retrospective	Qinghai Provincial People's Hospital (2010–2013)	AIHA (70/54)		✓	
Fu (2016)[39]	Retrospective	Tianjin Medical University General Hospital (2010–2015)	Patients with refractory AIHA (49/45)		✓	
Zhu (2016)[14]	Retrospective	Hospital (2010–2015)	AIHA (52/43)	✓		
Li (2015)[60]	Retrospective	Xiangya hospital (2003–2013)	AIHA (30/NR)		✓	✓
Yang (2014)[25]	Retrospective	Hospital of Soochow University (2010–2012)	AIHA after allo-HSCT (12/10)	✓	✓	
Zhang (2019)[42]	Retrospective	The Third Affiliated Hospital of Zhongshan University (2014–2018)	AIHA (60/NA)		✓	
Wang (2021)[61]	Prospective	Hospital of Southwest Medical University (2015–2018)	wAIHA (NA/84)		✓	

Author (Year)	Study type	Database/centre (study period)	Population (Sample size: AIHA/wAIHA)	Outcomes<br>Epidemiology	Clinical burden	Economic burden
Yu (2013)[62]	Retrospective	Center for Clinical Transfusion Medicine	AIHA (61/NA)		✓	
Gao (2013)[44]	Retrospective	Peking University People's Hospital	wAIHA (NA/65)		✓	
Yang (2020)[28]	Retrospective	Gansu Provincial People's Hospital (2014–2018)	AIHA (40/>50% had wAIHA)	✓	✓	
Zhao (2021)[37]	Prospective	General Hospital Tianjin University (2017–2019)	wAIHA (NA/12)		✓	
Xiao (2015)[41]	Prospective	Guizhou Provincial People's Hospital	wAIHA (NA/78)		✓	
India						
Das (2022)[63]	Retrospective	Hospital (2012–2021)	wAIHA (348/214)		✓	
Das (2021)[45]	Prospective	Apollo Gleneagles Hospital (2013–2019)	wAIHA (NA/229)		✓	
Ray (2021)[17]	Prospective	Sriram Chandra Bhanja Medical College and Hospital (2015–2018)	Primary AIHA (62/42)		✓	
Thailand						
Rattarittamrong (2016)[31]	Retrospective	(2002–2013)	wAIHA (NA/101)		✓	
Japan						
Kamesaki (2013)[23]	Prospective	Laboratory of the Center for Community Medicine (2003–2010)	AIHA (216/62)	✓		
Kamesaki (2021)[30]	Prospective	Center for Community Medicine laboratory (2015–2019)	Undiagnosed patients with AIHA (438/26)	✓		
Fu (2016)[39]	Retrospective	(2010–2015)	Adult patients with refractory AIHA (162/43)	✓		✓
Kamesaki (2017)[11]	NA	Evidence reported by the Japanese Ministry of Health (1998)	AIHA (NA)	✓		
South Korea						
Baek (2011)[24]	Retrospective	Chungnam National University Hospital (1994–2010)	Newly diagnosed patients with AIHA (32/25)<br>Patients were with primary wAIHA	✓		
Park (2016)[36]	Retrospective	Asan Medical Center (1989–1996)	Patients with SLE who performed a bone marrow study (9/NA)	✓		
Taiwan						

Author (Year)	Study type	Database/centre (study period)	Population (Sample size: AIHA/wAIHA)	Outcomes<br>Epidemiology	Clinical burden	Economic burden
Yen (2017)[12]	Retrospective	Taiwan Centres for Disease Control HIV Surveillance Database (2000–2012)	People aged ≥15 years living with HIV (30/NA)	✓		

Table S9. Statements not reaching consensus

Important screening tests aside from DAT
Solid phase agglutination
Microcolumn agglutination
Dual direct antiglobulin test to detect warm IgM
Indirect antiglobulin test (IAT) to detect IgG
Optimal strategies for evaluating and addressing the underlying diseases that cause secondary wAIHA
Both CT imaging and sIL-2R for lymphoid neoplasia, anti-nuclear antibodies and rheumatoid factor for collagen disease, asking about the medication for drug-induced wAIHA
Depending on the diseases, but a more extensive panel of autoimmune markers (including anti-extractable nuclear antigens) and exclusion of a lymphoproliferative disease by examination of the peripheral blood and bone marrow, and PET/CT scan, will be needed
The majority of the patients have SLE and are tested for AIHA and complement levels
Signs intervention is needed beyond "watch and wait"
Chronically low Hb and elevated bilirubin
Preferred treatment options in later lines of therapy for patients with primary wAIHA
Corticosteroids
Only splenectomy is covered by the public health insurance as secondary treatment for wAIHA.
(1) Rituximab (375 mg/m^{2}) intravenous infusion once weekly for a total of 4 doses (2) M-PSL (1,000 mg diluted in 250 ml of 5% glucose solution and administered as an intravenous infusion over 2 hours, once daily for 3 consecutive days, constituting one course) (3) Immunosuppressant: AZA (50 to 100 mg once daily after breakfast); Tacrolimus capsules (1 to 3 mg once daily, either after dinner or before breakfast); MMF (250 to 1,000 mg twice daily, given 12 hours apart); Cyclophosphamide (500–750 mg/m^{2} intravenous infusion every 4 weeks for a total of 6 doses) (4) Splenectomy
Short-term treatment goals for patients with wAIHA
Inhibit reticulocytosis and increase Hb to >10g/dL
Long-term treatment goals for patients with wAIHA
No statements
The most relevant clinical indicators to ensure appropriate follow-up and monitoring of patients with wAIHA
Hb level: monitor weekly or every two weeks
Hb, MCV, LDH, reticulocyte count, haptoglobin: monitor weekly to 6 monthly depending on tempo of disease at the moment;<br>DAT repeated only after milestones such as after achieving treatment-free status
Hb, LD, TB, IB, haptoglobin, reticulocytes, direct Coomb's test
Situations for considering blood transfusion for the management of wAIHA
Symptomatic anemia
First-line treatments
Rituximab (375 mg/m^{2} once a week for 4 weeks) + corticosteroids
Enrolment in clinical trials
Rituximab (1,000 mg given twice, 2 weeks apart) + corticosteroids
IVIG (1–2 g/kg, as a single infusion)
Second-line treatments (after failure)
Enrolment in clinical trials
Splenectomy
IVIG (1–2 g/kg, as a single infusion)
Rituximab (100 mg/week for 4 weeks) + corticosteroids
Second-line treatments after relapse
Splenectomy
IVIG (1–2 g/kg, as a single infusion)
Danazol (200–800 mg per day, divided into multiple doses)
Rituximab (100 mg/week for 4 weeks) + corticosteroids

Restart corticosteroids
MMF or CNI + initial course of steroid
Third- or later-line treatments after failure
Danazol (200–800 mg per day, divided into multiple doses)
IVIG (1–2 g/kg, as a single infusion)
Third- or later-line treatments after relapse
Danazol (200–800 mg per day, divided into multiple doses)
Rituximab (100 mg/week for 4 weeks)
First-line therapy for patients with wAIHA secondary to CTD
No statements
Later-line therapy for patients with wAIHA secondary to CTD
IVIG
Situations in which to consider treatment switch or add-on for patients with wAIHA
Time to wait before determining failure to respond: 4 weeks
Time to wait before determining failure to respond: 6 weeks
Definition of relapse: Worsening of anemia during cessation of the treatment
Definition of relapse: haemolysis after previously achieving normalisation of LDH/Hb/reticulocytes
Recurrence of anemia with positive DAT
Signs of "lack of response" in wAIHA
If Hb and haemolytic markers are not improved. Although normalisation of Hb is ideal, but the 2 mg/dL increase is accepted in real-world settings
Failure of Hb to increase to >10g/dL
Management of wAIHA comorbidities and potential complications (e.g., haemolysis-related organ dysfunction or thrombotic events)
Thrombosis
EPO, danazol, transfusions, iron infusions, low threshold to exclude DVT/PE
VTE prophylaxis, folic acid supplementation, anti-acid, PJP prophylaxis
If the haemolysis is mainly intravascular, use caution for renal damage and thrombosis. For renal damage, sufficient hydration would be necessary. For thrombosis, monitor D-dimer level, and take appropriate actions if necessary (such as anticoagulant).
Transfusion
Huge splenomegaly
Primary illness
The current challenges to diagnose wAIHA
No challenges, not difficult to diagnose wAIHA
Not sure if there are newer and most sensitive laboratory tests; DAT, reticulocyte count, haptoglobin, bilirubin and LDH are good enough
Reimbursement issue about rituximab
Lack of awareness
False negative of DAT
Heterogeneity of autoantibodies
Warm AIHA treatment gaps that need to be addressed with currently available options, including off-label treatments (e.g., immunosuppressants, immunoglobulins, rituximab, etc.)
Ongoing access and resources required for IVIG may be difficult. Limited options for refractory disease. Also, treatment indication for low grade (compensated) disease is unclear.
Access to danazol, access to EPO, access to clinical trials with novel agents, lack of awareness of thrombosis risk and mortality risk
There are only two treatments approved: corticosteroid and splenectomy
All immunosuppressants are off-label
Most treatments other than glucocorticoids are not covered by insurance for wAIHA
Challenges or barriers to overall management of wAIHA patients

Lack of appropriate referral pathway, and many wAIHA patients are on corticosteroids without trying another option (partly due to reimbursement policy)
Complications with blood transfusions
Further research and evidence-generation approaches that should be considered to address the evidence gaps in the published literature for wAIHA, specifically in the APAC region
Is wAIHA in SLE different to wAIHA in APLS? The latter is often harder to treat and more thrombotic complications.

Abbreviations: APAC: Asia-Pacific; APLS: antiphospholipid syndrome; AZA: azathioprine; CNI: calcineurin inhibitors; CT: computed tomography; CTD: connective tissue disease; DAT: direct antigen test; DVT/PE: deep vein thrombosis/pulmonary embolism; EPO: erythropoietin; Hb: hemoglobin; IAT: indirect antigen test; IB: indirect bilirubin; IgM: immunoglobulin M; IVIG: intravenous immunoglobulin; LDH: lactate dehydrogenase; MMF: mycophenol mofetil; PET: positron emission tomography; PJP: *Pneumocystis jirovecii* pneumonia; SLE: systemic lupus erythematosus; TB: total bilirubin; VTE: venous thromboembolism; wAIHA: warm autoimmune haemolytic anemia

Table S10. Statements with consensus disagreement

First-line treatments
Corticosteroid: dexamethasone (40 mg for 4 days every 4 weeks)
Second-line treatments after failure
Danazol (200–800 mg per day, divided into multiple dose)
Treatment considerations for secondary wAIHA
Probably not influenced by the underlying disease
Later-line therapy for patients with wAIHA secondary to CTD
TNF-alpha inhibitors
Challenges or barriers to overall management of wAIHA patients
Steroid is the only effective treatment option

Abbreviations: CTD: connective tissue disease; TNF: tumour necrosis factor; wAIHA: warm autoimmune haemolytic anemia

1.2 Appendix S2

Table S11. Summary of Delphi survey responses of part 1.1 Q1: what are the most important supplementary tests to consider during screening of patients, in addition to main test (direct antiglobulin test [DAT or Coombs test] to detect IgG + C3d or IgG) in current clinical guidelines?

Strategies provided by panellists in the first round	Proportion of panellists agreed	Consensus decision
Diagnostic tests		
Assessment for possible underlying diseases (e.g., CLL, cancer, SLE, infection, drug use, other autoimmune diseases) is important	8/9 (89%)	Agree, consensus reached
Solid phase agglutination is important	2/10 (20%)	No consensus
Microcolumn agglutination technique is important	3/10 (30%)	No consensus
Differential diagnosis tests		
Blood smear with spherocytes is important	8/9 (89%)	Agree, consensus reached
Dual direct antiglobulin test to detect warm IgM is important	7/10 (70%)	No consensus
Rheumatoid factor test in patients who might have rheumatoid arthritis is important	9/10 (90%)	Agree, consensus reached
Indirect antiglobulin test (IAT) to detect IgG is important	6/10 (60%)	No consensus

Abbreviations: CLL: chronic lymphocytic leukemia; IAT: indirect antigen test; IgG: immunoglobulin G; IgM: immunoglobulin M; SLE: systemic lupus erythematosus

Table S12. Summary of Delphi survey responses of part 1.1 Q2: what are the optimal strategies for evaluating and addressing the underlying diseases that cause secondary wAIHA?

Strategies provided by panellists in the first round	Proportion of panellists agreed	Consensus decision
Each rheumatic disease has its respective approach to evaluating disease activity and chronicity/ damage/ comorbidities of the individual	10/10 (100%)	Agree, consensus reached
Symptoms of possible underlying diseases and blood tests	9/10 (90%)	Agree, consensus reached
Rule out other hematologic disease	8/9 (88.9%)	Agree, consensus reached
Clinical review (history, examination, and relevant investigations) with a low threshold to exclude lymphoproliferative disease	9/10 (90%)	Agree, consensus reached
The most common initial step is to evaluate connective tissue disease	10/10 (100%)	Agree, consensus reached
To screen autoimmune diseases and malignancies	8/9 (89%)	Agree, consensus reached
Both CT imaging and sIL-2R for lymphoid neoplasia, anti-nuclear antibodies and rheumatoid factor for collagen disease, asking about the medication for drug-induced wAIHA	7/10 (70%)	No consensus
Depending on the diseases, but a more extensive panel of autoimmune markers (including anti extractable nuclear antigens) and exclusion of a lymphoproliferative disease by examination of the peripheral blood and bone marrow, and PET/CT scan, will be needed	7/10 (70%)	No consensus
The majority of the patients have SLE and are tested for AIHA and complement levels	7/10 (70%)	No consensus

Abbreviations: AIHA: autoimmune haemolytic anemia; CT: computed tomography; PET: positron emission tomography; sIL-2R: soluble interleukin-2 receptor; SLE: systemic lupus erythematosus; wAIHA: warm autoimmune haemolytic anemia

Table S13. Summary of Delphi survey responses of part 1.2 Q1: how you determine when intervention is needed versus “watch and wait” (i.e. what factors should be considered for treatment initiation in wAIHA)?

Strategies provided by panellists in the first round	Proportion of panellists agreed	Consensus decision
Hb level	9/9 (100%)	Agree, consensus reached
Symptoms of anemia / symptomatic anemia	9/9 (100%)	Agree, consensus reached
Severity of anemia	8/9 (89%)	Agree, consensus reached
Hb under 10 mg/dL, if the patients feel anaemic symptoms	10/10 (100%)	Agree, consensus reached
Trajectory of falling Hb	10/10 (100%)	Agree, consensus reached
Chronically low Hb; elevated bilirubin	7/10 (70%)	No consensus
Underlying diseases that cause wAIHA	9/10 (90%)	Agree, consensus reached

Abbreviations: Hb: hemoglobin; wAIHA: warm autoimmune haemolytic anemia

Table S14. Summary of Delphi survey responses of part 1.2 Q2: what are some of the preferrable treatment options in later lines (e.g., second line, third line, or after steroids) of therapy in patients with primary wAIHA? (Please specify dosing, frequency, and duration for each treatment)?

Strategies provided by panellists in the first round	Proportion of panellists agreed	Consensus decision
Immunosuppressant as second line	8/10 (80%)	Agree, consensus reached
Standard immunosuppressants such as AZA, IVIG, or rituximab	9/10 (90%)	Agree, consensus reached
Second line: IVIG, MMF, AZA, CNI	8/10 (80%)	Agree, consensus reached
Third line: rituximab	8/10 (80%)	Agree, consensus reached
Rituximab + steroids or MMF	8/10 (80%)	Agree, consensus reached
Corticosteroids	7/10 (70%)	No consensus
Rituximab	9/10 (90%)	Agree, consensus reached
Anti-CD20 antibodies	9/10 (90%)	Agree, consensus reached
Only splenectomy is covered by the public health insurance as secondary treatments for wAIHA. In Japanese guidelines, they mention about rituximab and other immunosuppressants but all of them are off label	4/10 (40%)	No consensus
(1) Rituximab 375 mg/m^2 intravenous infusion one weekly for a total of 4 doses (2) M-PSL 1,000 mg diluted in 250 mL of 5% glucose solution, administered as an intravenous infusion over 2 hours, once daily for 3 consecutive days, constituting one course (3) Immunosuppressant: AZA 50 to 100 mg once daily after breakfast; Tacrolimus capsules 1 to 3 mg once daily, either after dinner or before breakfast; MMF 250 to 1,000 mg twice daily, given 12 hours apart; CPA 500 to 750 mg/m^2 intravenous infusion every 4 weeks for a total of 6 doses (4) Splenectomy	7/10 (70%)	No consensus

Abbreviations: AZA: azathioprine; CNI: calcineurin inhibitors; CPA: cyclophosphamide; IVIG: intravenous immunoglobulin; M-PSL: methylprednisolone; MMF: mycophenol mofetil; wAIHA: warm autoimmune haemolytic anemia

Table S15. Summary of Delphi survey responses of part 1.2 Q3: what are the short-term and long-term treatment goals in patients with wAIHA?

Strategies provided by panellists in the first round	Proportion of panellists agreed	Consensus decision
Short-term treatment goals		
Improvement of anemia, Improvement in blood count, increase Hb	9/9 (100%)	Agree, consensus reached
Reduce symptomatic anemia	9/9 (100%)	Agree, consensus reached
Improve anaemic symptoms and tiredness	9/9 (100%)	Agree, consensus reached
Inhibit reticulocytosis and increase Hb to >10 g/dL	7/10 (70%)	No consensus
Stabilise Hb level	8/10 (80%)	Agree, consensus reached
Remission	9/10 (90%)	Agree, consensus reached
Long-term treatment goal		
Cessation of treatment	9/10 (90%)	Agree, consensus reached
Improve Hb and QOL, go back to work, to avoid side effects of corticosteroids	8/9 (89%)	Agree, consensus reached
Improvement of anemia (Hb level)	9/9 (100%)	Agree, consensus reached
Improvement of symptoms (fatigue, etc.)	8/9 (89%)	Agree, consensus reached
Minimise steroid burden	9/10 (90%)	Agree, consensus reached
Prevent/reduce relapse	10/10 (100%)	Agree, consensus reached
Stabilise disease status	8/10 (80%)	Agree, consensus reached
Extinguish active haemolysis without relying on long term toxic therapies	10/10 (100%)	Agree, consensus reached
Cessation of treatment with improvement of anemia	8/9 (89%)	Agree, consensus reached
Prevention of complications (infections, thrombosis, etc.)	10/10 (100%)	Agree, consensus reached

Abbreviations: Hb: hemoglobin; QOL: quality of life

Table S16. Summary of Delphi survey responses of part 1.2 Q4: what are the most relevant clinical indicators that could be used to ensure appropriate follow-up and monitoring of patients with wAIHA?

Strategies provided by panellists in the first round	Proportion of panellists agreed	Consensus decision
Hb level	8/9 (89%)	Agree, consensus reached
Haptoglobin and LDH<br>→Frequency depends on other comorbidities and if the patient has been admitted for other reasons, then weekly, otherwise it could be monthly or 2–3 monthly.	8/10 (80%)	Agree, consensus reached
Hb level, reticulocyte count, haptoglobin	10/10 (100%)	Agree, consensus reached
Hb level<br>→Frequency according to one panellist: follow up at least once every 3 months	8/10 (80%)	Agree, consensus reached
Hb level<br>→Frequency according to one panellist: follow up weekly or every two weeks	6/10 (60%)	No consensus
Hb, MCV, LDH, reticulocyte count, haptoglobin – Frequency: weekly to 6 monthly depending on tempo of disease at the moment; DAT – Frequency: repeated only after milestones such as after achieving treatment free status	7/10 (70%)	No consensus
Hb, LDH, and reticulocyte count<br>Frequency: If stable, every 2 months, if not, every month	9/10 (90%)	Agree, consensus reached
Hb, LDH, TB, IB, haptoglobin, reticulocytes, direct Coomb's test	4/10 (40%)	No consensus

Abbreviations: DAT: direct antigen test; Hb: hemoglobin; IB: indirect bilirubin; LDH: lactate dehydrogenase; TB: total bilirubin

Table S17. Summary of Delphi survey responses of part 1.2 Q5: in what cases should blood transfusion be considered for management in wAIHA, considering the potential challenges associated with alloimmunisation and delay of haemolytic reaction?

Strategies provided by panellists in the first round	Proportion of panellists agreed	Consensus decision
Hb <7 g/dL or <9 g/dL if there is comorbid ischemic heart disease	8/9 (89%)	Agree, consensus reached
Severe symptomatic anemia (Hb <5g/dL)	9/10 (90%)	Agree, consensus reached
Severe anemia (Hb <6g/dL)	8/10 (80%)	Agree, consensus reached
Hb < 7g/dL and symptom	8/9 (89%)	Agree, consensus reached
Hb is <7 g/dL and moderate and severe anemic symptoms. It depends on the age and comorbidities such as chronic heart failure. I am not worrying about alloimmunisation, as its incidence is not so high	9/9 (100%)	Agree, consensus reached
Severe symptomatic anemia, refractory to steroids	9/10 (90%)	Agree, consensus reached
Symptomatic anemia	4/10 (40%)	No consensus
Life-threatening condition	10/10 (100%)	Agree, consensus reached
Planned surgery	9/10 (90%)	Agree, consensus reached
Severe complications	9/10 (90%)	Agree, consensus reached

Abbreviations: Hb: hemoglobin

Table S18. Summary of Delphi survey responses of part 1.2 Q6: treatment options and preferences in wAIHA per treatment line and after failure or relapse.

Strategies provided by panellists in the first round	Proportion of panellists agreed	Consensus decision	Proportion of panellists, preferred choice	Consensus decision2
First-line treatments				
Corticosteroid: prednisone (1mg/kg daily for 3 weeks)	9/9 (100%)	Agree, consensus reached	9/9 (100%)	Agree, consensus reached
Corticosteroid: intravenous methylprednisolone (250–1000 mg per day for 1–3 days)	8/10 (80%)	Agree, consensus reached	4/10 (40%)	No consensus
Rituximab (375 mg/m² once a week for 4 weeks) + corticosteroids	7/10 (70%)	No consensus	5/10 (50%)	No consensus
Enrolment into clinical trials	3/10 (30%)	No consensus	2/10 (20%)	Disagree, consensus reached
Rituximab (1,000 mg given twice, 2 weeks apart) + corticosteroids	6/10 (60%)	No consensus	3/10 (30%)	No consensus
Intravenous immunoglobulin (IVIG) (1–2 g/kg, as a single infusion)	0/10 (0%)	Disagree, consensus reached	0/10 (0%)	Disagree, consensus reached
Corticosteroid: dexamethasone (40 mg for 4 days every 4 weeks)	1/10 (10%)	Disagree, consensus reached	0/10 (0%)	Disagree, consensus reached
Second line treatments (after failure)				
Immunosuppressants	9/9 (100%)	Agree, consensus reached	8/9 (89%)	Agree, consensus reached
Cyclosporin (2–5 mg/kg per day split into two doses)	8/9 (89%)	Agree, consensus reached	7/10 (70%)	No consensus
Azathioprine (1–2 mg/kg/day split into multiple doses)	8/9 (89%)	Agree, consensus reached	7/10 (70%)	No consensus
Enrolment into clinical trials	7/10 (70%)	No consensus	6/10 (60%)	No consensus
Splenectomy	5/10 (50%)	No consensus	1/10 (10%)	Disagree, consensus reached

Splenectomy	5/10 (50%)	No consensus	1/10 (10%)	Disagree, consensus reached
Danazol (200–800 mg per day, divided into multiple dose)	1/10 (10%)	Disagree, consensus reached	1/10 (10%)	Disagree, consensus reached
IVIG (1–2 g/kg, as a single infusion)	5/10 (50%)	No consensus	3/10 (30%)	No consensus
Rituximab (375 mg/m² once a week for 4 weeks)	9/10 (90%)	Agree, consensus reached	6/10 (60%)	No consensus
Rituximab (1,000 mg given twice, 2 weeks apart) + corticosteroids	8/10 (80%)	Agree, consensus reached	6/10 (60%)	No consensus
Rituximab (1,000 mg given twice, 2 weeks apart) + corticosteroids	8/10 (80%)	Agree, consensus reached	6/10 (60%)	No consensus
Rituximab (fixed dose of 1,000 mg given twice, 2 weeks apart)	8/10 (80%)	Agree, consensus reached	5/10 (50%)	No consensus
Rituximab (375 mg/m² once a week for 4 weeks) + corticosteroids	8/10 (80%)	Agree, consensus reached	6/10 (60%)	No consensus
Rituximab (fixed dose of 100mg/week for 4 weeks) + corticosteroids	2/10 (20%)	Disagree, consensus reached	2/10 (20%)	Disagree, consensus reached
Second line treatments (after relapse)				
Immunosuppressants	8/9 (89%)	Agree, consensus reached	7/10 (70%)	No consensus
Cyclosporin (2–5 mg/kg per day split into two doses)	9/9 (100%)	Agree, consensus reached	7/10 (70%)	No consensus
Enrolment into clinical trials	8/10 (80%)	Agree, consensus reached	7/10 (70%)	No consensus
Azathioprine (1–2 mg/kg/day split into multiple doses)	10/10 (100%)	Agree, consensus reached	6/10 (60%)	No consensus
Splenectomy	5/10 (50%)	No consensus	0/10 (0%)	Disagree, consensus reached

IVIG (1–2 g/kg, as a single infusion)	6/10 (60%)	No consensus	3/10 (30%)	No consensus
Rituximab (375 mg/m² once a week for 4 weeks) + corticosteroids	10/10 (100%)	Agree, consensus reached	7/10 (70%)	No consensus
Splenectomy	5/10 (50%)	No consensus	0/10 (0%)	Disagree, consensus reached
Danazol (200–800 mg per day, divided into multiple dose)	2/10 (20%)	Disagree, consensus reached	1/10 (10%)	Disagree, consensus reached
Rituximab (375 mg/m² once a week for 4 weeks)	9/10 (90%)	Agree, consensus reached	5/10 (50%)	No consensus
Rituximab (fixed dose of 1,000 mg given twice, 2 weeks apart)	9/10 (90%)	Agree, consensus reached	5/10 (50%)	No consensus
Rituximab (1,000 mg given twice, 2 weeks apart) + corticosteroids	9/10 (90%)	Agree, consensus reached	6/10 (60%)	No consensus
Rituximab (fixed dose of 100mg/week for 4 weeks) + corticosteroids	5/10 (50%)	No consensus	3/10 (30%)	No consensus
Restart corticosteroids	7/10 (70%)	No consensus	4/10 (40%)	No consensus
Switch to MMF or CNIs for relapse plus initial course of steroid	6/10 (60%)	No consensus	3/10 (30%)	No consensus
Third or later line treatments (after failure)				
Enrolment into clinical trials	10/10 (100%)	Agree, consensus reached	7/10 (70%)	No consensus
Splenectomy	10/10 (100%)	Agree, consensus reached	3/10 (30%)	No consensus
MMF	8/9 (89%)	Agree, consensus reached	8/9 (89%)	Agree, consensus reached
Cyclophosphamide (1–2 mg/kg per day)	10/10 (100%)	Agree, consensus reached	5/10 (50%)	No consensus
Danazol (200–800 mg per day, divided into multiple dose)	6/10 (60%)	No consensus	2/10 (20%)	Disagree, consensus reached

IVIG (1–2 g/kg, as a single infusion)	4/10 (40%)	No consensus	1/10 (10%)	Disagree, consensus reached
Rituximab (375 mg/m² once a week for 4 weeks)	9/9 (100%)	Agree, consensus reached	8/9 (89%)	Agree, consensus reached
Rituximab (fixed dose of 1,000 mg given twice, 2 weeks apart)	9/10 (90%)	Agree, consensus reached	5/10 (50%)	No consensus
Third or later line treatments (after relapse)				
Enrolment into clinical trials	8/9 (89%)	Agree, consensus reached	8/9 (89%)	Agree, consensus reached
Splenectomy	9/9 (100%)	Agree, consensus reached	8/9 (89%)	Agree, consensus reached
Cyclophosphamide (1–2 mg/kg per day)	9/10 (90%)	Agree, consensus reached	4/10 (40%)	No consensus
MMF	8/9 (89%)	Agree, consensus reached	6/10 (60%)	No consensus
Danazol (200–800 mg per day, divided into multiple dose)	6/10 (60%)	No consensus	2/10 (20%)	Disagree, consensus reached
Rituximab (375 mg/m² once a week for 4 weeks)	8/9 (89%)	Agree, consensus reached	7/10 (70%)	No consensus
Rituximab (fixed dose of 100mg/week for 4 weeks)	7/10 (70%)	No consensus	5/10 (50%)	No consensus
Rituximab (fixed dose of 1,000 mg given twice, 2 weeks apart)	8/10 (80%)	Agree, consensus reached	6/10 (60%)	No consensus

Abbreviations: CNI: calcineurin inhibitors; IVIG: intravenous immunoglobulin; MMF: mycophenol mofetil

Table S19. Summary of Delphi survey responses of part 1.2 Q7: regarding will the treatment options be affected by the underlying disease in cases of secondary wAIHA? What are the most effective treatment options in first line of therapy in patients with wAIHA secondary to connective tissue diseases (CTD)? What are the most effective treatment options in later lines of therapy in patients with wAIHA secondary to connective tissue diseases (CTD)?

Strategies provided by panellists in the first round	Proportion of panellists agreed	Consensus decision
Will the treatment options be affected by the underlying disease in cases of secondary wAIHA?		
Yes, Treatment of underlying disease is the first choice	9/9 (100%)	Agree, consensus reached
Probably not	0/10 (0%)	Disagree, consensus reached
Yes, Lower dose of steroid plus MMF combination	1/10 (10%)	Disagree, consensus reached
Yes, Treatment of underlying diseases may lead to a remission of the AIHA	10/10 (100%)	Agree, consensus reached
Yes, Treatment directed towards the underlying disease (e.g. lymphoma, CLL, inflammatory bowel disease, etc.)	8/9 (89%)	Agree, consensus reached
Comorbidity	8/10 (80%)	Agree, consensus reached
Yes, in that case, immunosuppressant would become a lower priority	2/10 (20%)	Disagree, consensus reached
Yes. We treat the underlying disease first. For instance, PSL for SLE, chemotherapy for lymphoid malignancies, stop medicine for drug-induced wAIHA.	8/9 (89%)	Agree, consensus reached
Most effective first-line options for treatment of wAIHA secondary to CTD		
Corticosteroids	9/9 (100%)	Agree, consensus reached
Steroid and MMF	9/10 (90%)	Agree, consensus reached
Treat connective tissue disease first	9/10 (90%)	Agree, consensus reached
Most effective later-line options for treatment of wAIHA secondary to CTD		
Rituximab	8/9 (89%)	Agree, consensus reached
IVIG	3/10 (30%)	No consensus
Immunosuppressants	10/100 (100%)	Agree, consensus reached
CNI	9/10 (90%)	Agree, consensus reached

Steroid pulse	9/9 (100%)	Agree, consensus reached
Corticosteroid + Rituximab	8/10 (80%)	Agree, consensus reached
MMF	10/10 (100%)	Agree, consensus reached
TNF-alpha inhibitors	0/10 (0%)	Disagree, consensus reached

Abbreviations: AIHA: autoimmune haemolytic anemia; CLL: chronic lymphocytic leukemia; CNI: calcineurin inhibitors; CTD: connective tissue disease; MMF: mycophenol mofetil; PSL: prednisolone; SLE: systemic lupus erythematosus; TNF: tumour necrosis factor; wAIHA: warm autoimmune haemolytic anemia

Table S20. Summary of Delphi survey responses of part 1.2 Q8: when to consider treatment switch or add-on for patients with wAIHA?

Strategies provided by panellists in the first round	Proportion of panellists agreed	Consensus decision
Lack of response	9/9 (100%)	Agree, consensus reached
Time to wait before determining failure to respond: 4 weeks	6/10 (60%)	No consensus
Time to wait before determining failure to respond: 6 weeks	6/10 (60%)	No consensus
Time to wait depends on the drugs, e.g., corticosteroids expect rapid response every day, whereas rituximab we expect over months	8/10 (80%)	Agree, consensus reached
Relapse	8/9 (89%)	Agree, consensus reached
Definition of relapse: drop in Hb again to <10g/dL due to haemolysis	8/10 (80%)	Agree, consensus reached
Definition of relapse: worsening of anemia during cessation of the treatment	5/10 (50%)	No consensus
Definition of relapse: after initial response and then deterioration of Hb supported by evidence of active haemolysis	9/10 (90%)	Agree, consensus reached
Definition of relapse: haemolysis after previously achieving normalisation of LDH, Hb, and reticulocytes	7/10 (70%)	No consensus
Recurrence of anemia with positive DAT	7/10 (70%)	No consensus
Recurrence	7/8 (88%)	Agree, consensus reached
Definition of recurrence: same as relapse	9/9 (100%)	Agree, consensus reached
Complications	7/8 (88%)	Agree, consensus reached
Patient preference	7/8 (88%)	Agree, consensus reached
What is meant by 'lack of response'?		
Still transfusion dependent	8/9 (89%)	Agree, consensus reached
No parameter change (Hb, LDH, reticulocytes, etc.) in 4 weeks	9/10 (90%)	Agree, consensus reached
If Hb and haemolytic markers are not improved. Although normalisation of Hb is ideal, but the 2 mg/dL increase is accepted by real world settings	6/10 (60%)	No consensus
Failure of Hb to increase to >10g/dL	6/10 (60%)	No consensus
Hb levels, symptoms of anemia	8/9 (89%)	Agree, consensus reached

Abbreviations: DAT: direct antigen test; Hb: hemoglobin; LDH: lactate dehydrogenase

Table S21. Summary of Delphi survey responses of part 1.2 Q9: how do you manage the primary concerns in the management of comorbidities and potential complications associated with wAIHA, such as haemolysis-related organ dysfunction or thrombotic events?

Strategies provided by panellists in the first round	Proportion of panellists agreed	Consensus decision
Appropriate management of complications	8/9 (89%)	Agree, consensus reached
Minimised steroid toxicities	8/9 (89%)	Agree, consensus reached
Minimise steroid use and early combination with MMF or AZA	9/10 (90%)	Agree, consensus reached
Thrombosis	5/10 (50%)	No consensus
EPO, danazol, transfusions, iron infusions, low threshold to exclude DVT/PE	3/10 (30%)	No consensus
VTE prophylaxis, folic acid supplementation, anti-acid, PJP prophylaxis	6/10 (60%)	No consensus
If the haemolysis is mainly intravascular, use caution for renal damage and thrombosis. For renal damage, sufficient hydration would be necessary. For thrombosis, monitor D-dimer level, and take appropriate actions if necessary (such as anticoagulant).	6/10 (60%)	No consensus
Increase Hb to avoid heart failure. If the patients experienced thrombotic events such as brain infarction and ischemic heart disease, we prescribe anti-platelet medicine	9/10 (90%)	Agree, consensus reached
Transfusion	7/10 (70%)	No consensus
Huge splenomegaly	3/10 (30%)	No consensus
Adverse effects of glucocorticosteroids and immunosuppressants	9/10 (90%)	Agree, consensus reached
Primary illness	6/10 (60%)	No consensus

Abbreviations: AZA: azathioprine; DVT/PE: deep vein thrombosis/pulmonary embolism; EPO: erythropoietin; MMF: mycophenol mofetil; PJP: *Pneumocystis jirovecii* pneumonia; VTE: venous thromboembolism

Table S22. Summary of Delphi survey responses of part 2 Q1: what are the current challenges to diagnose wAIHA patients?

Strategies provided by panellists in the first round	Proportion of panellists agreed	Consensus decision
No challenges, not difficult to diagnose wAIHA	4/10 (40%)	No consensus
Not sure if there are newer and most sensitive laboratory tests; the current DAT, reticulocyte count, haptoglobin, bilirubin and LDH are good enough	5/10 (50%)	No consensus
Steroid complications	10/10 (100%)	Agree, consensus reached
Reimbursement issue about rituximab	7/10 (70%)	No consensus
Symptomatic anemia are generally well captured in Japan, but differential diagnosis in AIHA (wAIHA, CAD, or mixed AIHA) is sometimes challenging. Also, Coombs negative wAIHA is often difficult to be diagnosed	9/10 (90%)	Agree, consensus reached
Low disease awareness of physicians and patients. As some of wAIHA patients have low titer antibody, we need more sensitive diagnostic tests	8/10 (80%)	Agree, consensus reached
Lack of awareness	7/10 (70%)	No consensus
Heterogeneity of disease manifestations	8/10 (80%)	Agree, consensus reached
False negative of DAT	6/10 (60%)	No consensus
Heterogeneity of autoantibodies	4/10 (40%)	No consensus
Lack of standardised guidelines	10/10 (100%)	Agree, consensus reached

Abbreviations: AIHA: autoimmune haemolytic anemia; CAD: cold agglutinin disease; DAT: direct antigen test; LDH: lactate dehydrogenase; wAIHA: warm autoimmune haemolytic anemia

Table S23. Summary of Delphi survey responses of part 2 Q2: what are the current gaps in the treatment of wAIHA that need to be addressed with current available options, including off-label treatments (e.g., immunosuppressants, immunoglobulins, rituximab, etc.) in your country?

Strategies provided by panellists in the first round	Proportion of panellists agreed	Consensus decision
Rituximab is not approved	9/10 (90%)	Agree, consensus reached
Ongoing access and resources required for IVIG may be difficult. Limited options for refractory disease. Also, treatment indication for low grade (compensated) disease is unclear.	5/10 (50%)	No consensus
Rituximab is expensive – earlier use may reduce relapse rate; no other biological agents shown to be effective at this moment	8/10 (80%)	Agree, consensus reached
Access to danazol, access to EPO, access to clinical trials with novel agents, lack of awareness of thrombosis risk and mortality risk	3/10 (30%)	No consensus
There are only two treatments approved: corticosteroid and splenectomy	5/10 (50%)	No consensus
All of immunosuppressants are off label in your country	6/10 (60%)	No consensus
Absence of an evidence-based algorithm due to lack of high-quality clinical trials	10/10 (100%)	Agree, consensus reached
Most treatments other than glucocorticosteroids are not covered by insurance for wAIHA (rituximab, tacrolimus, MMF, etc.)	6/10 (60%)	No consensus

Abbreviations: EPO: erythropoietin; IVIG: intravenous immunoglobulin; MMF: mycophenol mofetil; wAIHA: warm autoimmune haemolytic anemia

Table S24. Summary of Delphi survey responses of part 2 Q3: what are the current challenges in or barriers to overall management of wAIHA patients?

Strategies provided by panellists in the first round	Proportion of panellists agreed	Consensus decision
Absence of an evidence-based algorithm due to lack of high-quality clinical trials	8/9 (89%)	Agree, consensus reached
Treatments not being reimbursed in their country	8/10 (80%)	Agree, consensus reached
Duration of maintenance immunosuppression is not clear, multiple team involvement can be confusing	10/10 (100%)	Agree, consensus reached
Refractory disease difficult to manage	10/10 (100%)	Agree, consensus reached
Steroid is only effective treatment option<br>→ Answer from a panellist in Korea	1/10 (10%)	Disagree, consensus reached
Unpredictable relapse, low awareness of mortality risk	10/10 (100%)	Agree, consensus reached
Lack of appropriate referral pathway, and many wAIHA patients are on corticosteroids without trying another option (partly due to reimbursement policy)	5/10 (50%)	No consensus
Lack of awareness about the side effects of PSL for physicians. Off-label of rituximab and immunosuppressants, most haematologists believe Hb 7 is acceptable for wAIHA patients, although they are anaemic. 90% are oncologists and under 10% are haematologists, so most of wAIHA patients see oncologists who do not have enough knowledge and experience<br>→ Answer from a panellist in Japan	8/10 (80%)	Agree, consensus reached
Complications with blood transfusions	4/10 (40%)	No consensus
Development of new therapies	10/10 (100%)	Agree, consensus reached

Abbreviations: Hb: haemoglobin; PSL: prednisolone; wAIHA: warm autoimmune haemolytic anemia

Table S25. Summary of Delphi survey responses of part 2 Q4: what potential further research and evidence generation approaches should be considered to address the evidence gaps in the published literature for wAIHA, specifically in the APAC region?

Strategies provided by panellists in the first round	Proportion of panellists agreed	Consensus decision
Is wAIHA in SLE different to wAIHA in APLS? The latter is often harder to treat and more thrombotic complications.	4/10 (40%)	No consensus
Newer biological therapies to reduce the need to resort to splenectomy	9/9 (100%)	Agree, consensus reached
What causes poor prognosis in wAIHA in AP region? Information about comorbidities of wAIHA (thrombosis, malignancies etc) is lacking	10/10 (100%)	Agree, consensus reached
New treatment option except steroid is very promising	10/10 (100%)	Agree, consensus reached
Incidence and outcomes in the post-rituximab era	8/10 (80%)	Agree, consensus reached
A drug that has proven effectiveness through clinical trials is needed	8/9 (89%)	Agree, consensus reached
Medical advisor boards, Scientific meetings, comparing guidelines and real clinical practice in each country, registration of wAIHA, survey the national and health insurance data base	10/10 (100%)	Agree, consensus reached
Clinical trials comparing corticosteroids with other agents in the first-line setting	10/10 (100%)	Agree, consensus reached
Clinical trials comparing various second-line options	10/10 (100%)	Agree, consensus reached
Assessment of QOL for wAIHA patients	8/8 (100%)	Agree, consensus reached
Awareness about wAIHA among healthcare professionals	10/10 (100%)	Agree, consensus reached

Abbreviations: AP: Asia-Pacific; APLS: antiphospholipid syndrome; QOL: quality of life; SLE: systemic lupus erythematosus; wAIHA: warm autoimmune haemolytic anemia
